# Combining DNMT and HDAC6 inhibitors increases anti-tumor immune signaling and decreases tumor burden in ovarian cancer

**DOI:** 10.1038/s41598-020-60409-4

**Published:** 2020-02-26

**Authors:** Sara Moufarrij, Aneil Srivastava, Stephanie Gomez, Melissa Hadley, Erica Palmer, Paul Tran Austin, Sarah Chisholm, Noor Diab, Kyle Roche, Angela Yu, Jing Li, Wenge Zhu, Micael Lopez-Acevedo, Alejandro Villagra, Katherine B. Chiappinelli

**Affiliations:** 1 The George Washington University Cancer Center, The George Washington University, Washington, DC USA; 2 The Department of Obstetrics & Gynecology, The George Washington University, Washington, DC USA; 3 The Department of Microbiology, Immunology, & Tropical Medicine, The George Washington University, Washington, DC USA; 4 The Department of Biochemistry and Molecular Medicine, The George Washington University, Washington, DC USA; 5 The Institute for Biomedical Sciences, The George Washington University, Washington, DC 20052 USA

**Keywords:** Immunoediting, DNA methylation

## Abstract

Novel therapies are urgently needed for ovarian cancer, the deadliest gynecologic malignancy. Ovarian cancer has thus far been refractory to immunotherapies that stimulate the host immune system to recognize and kill cancer cells. This may be because of a suppressive tumor immune microenvironment and lack of recruitment and activation of immune cells that kill cancer cells. Our previous work showed that epigenetic drugs including DNA methyltransferase inhibitors and histone deacetylase 6 inhibitors (DNMTis and HDAC6is) individually increase immune signaling in cancer cells. We find that combining DNMTi and HDAC6i results in an amplified type I interferon response, leading to increased cytokine and chemokine expression and higher expression of the MHC I antigen presentation complex in human and mouse ovarian cancer cell lines. Treating mice bearing ID8 Trp53−/− ovarian cancer with HDAC6i/DNMTi led to an increase in tumor-killing cells such as IFNg+ CD8, NK, and NKT cells and a reversal of the immunosuppressive tumor microenvironment with a decrease in MDSCs and PD-1^hi^ CD4 T cells, corresponding with an increase in survival. Thus combining the epigenetic modulators DNMTi and HDAC6i increases anti-tumor immune signaling from cancer cells and has beneficial effects on the ovarian tumor immune microenvironment.

## Introduction

The five-year survival for ovarian cancer has remained unchanged for decades, and novel therapies are urgently needed^1^. Ovarian cancer has the deadliest outcome among gynecologic cancers due to its late (typically Stage III or IV) presentation and aggressive phenotype. High-grade serous ovarian cancer, the most common subtype, is characterized by genomic instability with >95% of cases exhibiting mutations in the tumor suppressor P53^2^. Treatment involves surgical staging and optimal debulking to reduce tumor burden, followed by chemotherapy with a platinum-based agent and a taxane, or vice versa (chemotherapy before surgery). Unfortunately most cancers recur within two years of chemotherapy. Significant advances in the pathobiology of the disease, including the identification of a subset of tumors with defects in homologous recombination (HR), have led to the use of PARP inhibitors, which can cause synthetic lethality in HR-deficient tumors. However, less than half of ovarian cancers are HR-deficient and there is no curative therapy for the majority of ovarian cancer patients^1,3^.

Cancer cells may be recognized as foreign by host immune cells that kill the cancer cells, but as they progress cancers exhibit mechanisms of immune evasion or immunoediting^4^. The tumor microenvironment (TME) is composed of both pro- and anti- cancer immune cells. These include CD8 effector T cells that recognize specific antigens on tumor cells to kill them, natural killer (NK) cells, part of the innate immune system that can kill tumor cells, and immuno-suppressive cell types including macrophages, regulatory T cells, and myeloid-derived suppressor cells. Novel drugs that activate CD8 effector T cells to fight cancer cells, including the checkpoint blockade inhibitors anti-PD-1 and anti-CTLA-4, have shown encouraging results in solid tumors^5,6^. Tumor cells can express programmed cell death ligand-1 (PD-L1), a receptor that binds to programmed cell death protein 1 (PD-1) on T cells, suppressing activated T cells. Cytotoxic T lymphocytes express CTLA-4 (cytotoxic T-lymphocyte associated protein 4), which binds to CD80 on antigen-presenting cells, decreasing their priming activity on T cells and causing cell cycle arrest^4,6^. While blockade of the PD-1 and CTLA-4 checkpoints has shown vigorous and durable responses in tumors such as melanoma and non-small cell lung cancer, it has not shown similar success in ovarian cancer with response rates less than 10%^7^. Response to immune therapy often correlates with the level of tumor infiltrating lymphocytes (TILs) and the number of tumor neoantigens present in tumors^6^; ovarian cancer has a low neoantigen burden and few TILs^1^.

Therapies that impact epigenetic regulation in cancer can have immunomodulatory effects. Cancers exhibit changes in the silencing DNA methylation mark (addition of a methyl group to the cytosines of CpG dinucleotides by DNA methyltransferases or DNMTs) compared to normal cells, including global loss of methylation at regions silenced for genome stability and gain of methylation at promoter regions of tumor suppressor genes^8^. 5-azacytidine (Aza) is a cytidine analog that inhibits DNMTs, triggering the re-expression of genes silenced by DNA methylation^8^. The DNMT inhibitors Aza and 5-aza-2-deoxycytidine (Dac) are FDA approved for the treatment of myelodysplastic syndrome^9^. We^10–14^ and others^15–18^ have shown that DNMTis upregulate immune signaling, including the interferon response, tumor-associated antigens (TAAs), and antigen presentation, in ovarian cancer and other solid tumors. DNMTis increase MHC Class I antigen presentation to immune cells^11,16–18^. DNMTis activate the canonical interferon (IFN) signaling pathway through upregulation of double-stranded RNA (dsRNA) from repetitive elements (REs), specifically hypermethylated endogenous retroviruses (ERVs) that activate dsRNA sensors TLR3 and MDA5^14,19^. REs are silenced by DNA methylation in somatic cells but frequently lose methylation in primary tumors and may be reactivated^20–28^. Treating a mouse model of OC with DNMTi activates ERVs, brings in T Effector cells, and reduces tumor burden in an IFN-dependent manner^12^. ERVs themselves can translate proteins that may be targeted as tumor-associated antigens^29^. Thus RE activation both promotes interferon signaling^30^ and presents potential tumor-specific antigens as T cell targets. Interestingly, DNMTi have been shown to increase methylation of a subset of genes in ovarian cancer and thus may also affect immune regulatory pathways by increasing methylation^31^.

Besides DNA methylation, modifications of histone proteins can also alter immune gene expression. Histone deacetylase (HDAC) enzymes remove acetyl groups from histones to decrease gene transcription. The hypo-acetylated positively charged histones remain tightly bound to the negatively charged DNA, closing the chromatin and preventing transcription^8,32^. In addition to histones, HDACs can deacetylate non-histone proteins to modify their function^32^. HDACs are divided into four classes: class I (HDACs 1, 2, 3, and 8) are found in the nucleus; class II (HDACs 4,5,6,7,9, and 10) are found in both the nucleus and cytoplasm; and class IV (HDAC11) is both nuclear and cytoplasmic. Class III HDACs are made up of sirtuins (SIRT1–7) and have a different mechanism of action, using NAD+ rather than Zn2+ as a cofactor^33^. Inhibiting HDACs reduces tumor proliferation and several pan-HDAC inhibitors are approved for the treatment of T cell lymphomas^33^.

The Class II HDAC HDAC6 is overexpressed in several cancer types, including ovarian cancer, prostate cancer, and acute myeloid leukemia^34^. HDAC6 is critical for modulation of immune responses^35–38^. HDAC6 promotes STAT3 signaling, inhibits antigen presentation, and upregulates immunosuppressive ligands^35,37,39,40^. Thus, inhibiting HDAC6 has the potential to increase anti-tumor immunity. The HDAC6i used in this study, Nexturastat A (NextA), augments immunotherapies including anti-PD-1. In a melanoma mouse model, the combination therapy significantly decreased tumor burden over either HDAC6i or anti-PD-1 treatments, increased cytotoxic T cells in the TME, and reduced the anti-inflammatory properties of tumor associated macrophages^37^. The HDAC6i ACY1215 improved survival in mice with ARID1A mutated ovarian clear cell carcinoma^41^ and ACY1215 combined with anti-PD-L1 immune checkpoint blockade in ARID1A mutated murine ovarian clear cell carcinoma reduced tumor burden and improved survival^42^.

While DNMTis have shown success in resensitizing carboplatin-resistant ovarian cancers^43–45^, combining DNMTis with HDAC6i is a novel approach for ovarian cancer. We hypothesized that combining both epigenetic modulators could reverse the immunosuppressive tumor microenvironment. Our findings show that combining HDAC6i and DNMTi upregulates interferon signaling, antigen presentation, and cytokines more so than each epigenetic drug alone in ovarian cancer cell lines. HDAC6i plus DNMTi treatment in a mouse model of ovarian cancer leads to reversal of the immunosuppressive TME. As both epigenetic therapies are currently in clinical trials in combination with immune therapy, this work has significant translational implications.

## Results

### Ovarian cancer cell lines are sensitive to HDAC6 inhibitors

**Figure 1:**
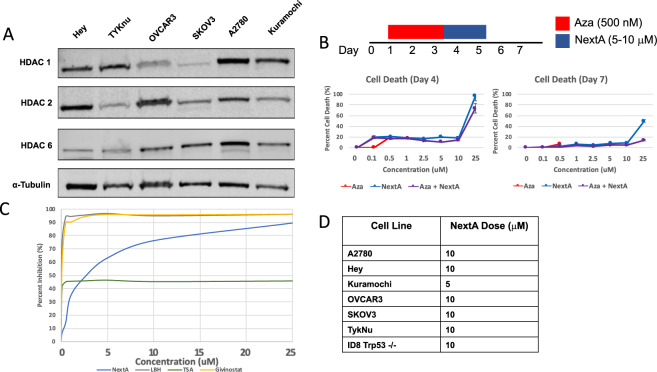
Ovarian cancer cell lines are sensitive to HDAC6 inhibitors. (**A**) Baseline levels of HDAC protein vary between cell lines. Protein was isolated from six different human ovarian cancer cell lines and levels of HDACs 1, 2, and 6 were assessed. α-tubulin was used as a loading control. HDAC1 and HDAC6 were run on the same blot which was cut to image, then stripped and reprobed for HDAC2 and stripped and reprobed for α-tubulin. Cropped blots are shown here and black lines indicate where one part of the blot ends and another begins. Figure S7A shows the entire blot images. (**B**) The Hey cell line was treated with indicated concentrations of the HDAC6 inhibitor Nexturastat A (NextA) and 500 nM of the DNMT inhibitor 5-azacytidine (Aza) in the following treatment schema: 3 days of Aza treatment followed by 2 days of NextA treatment. Cytotoxicity was assessed at Days 4 and 7 using the CellTox Green cytotoxicity assay, which quantitatively detects cell death. (**C**) The Hey cell line was treated with indicated concentrations of NextA and broader spectrum HDAC inhibitors Givinostat (Class I & Class II HDACi), LBH (pan-HDACi), TSA (pan-HDACi). The percent inhibition of HDAC activity was calculated using the HDAC-Glo assay. (**D**) The optimal NextA dose for each cell line was chosen as the dose that inhibited greater than 50% of HDAC activity while not causing more than 20% cytotoxicity.

To determine whether HDAC6 inhibition is a viable therapeutic strategy for ovarian cancer, we measured the protein levels of HDACs 1, 2, and 6 in human ovarian cancer cell lines (Fig. 1A). We observed significant variation in the protein levels of HDACs 1, 2, and 6 in the six ovarian cancer cell lines tested (Fig. 1A). For each cell line, we chose the dose of the HDAC6i Nexturastat A (“NextA”) that inhibited 70% of HDAC6 activity while causing no more than 20% cell death (Fig. 1B–D; Fig. S1; 5–10 uM). The pan-HDAC inhibitors LBH, TSA, and Givinostat are shown as positive controls for HDAC inhibition (Figs. 1C, S2). These pan-HDAC inhibitors are able to inhibit 100% of HDAC activity even at low doses (250–500 nM) and are included as positive controls. The HDAC6 specific inhibitor NextA inhibits between 60–80% of HDAC activity at 5 and 10 uM concentrations (Fig. 1C,D).

### Combining DNMTi and HDAC6i increases the Type I interferon response and cytokine expression

**Figure 2:**
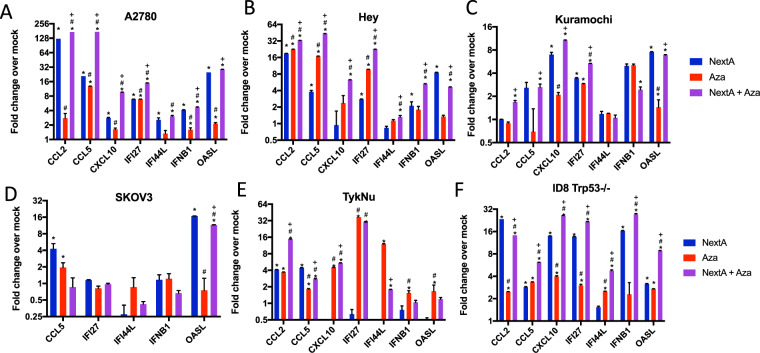
DNMTi and HDAC6i increase the expression of Interferon Stimulated Genes (ISGs) and cytokines. RNA was isolated from ovarian cancer cell lines at Day 7 of the treatment schema in Fig. 1 and qRT-PCR was performed for ISGs *IFI27, OASL, IFI44L*, and *IFNB1* and cytokines *CCL2, CCL5, and CXCL10*. Fold change is indicated relative to Mock for each of the following treatments: NextA (blue), Aza (red), and NextA+ Aza (purple) in the following cell lines: (**A**) A2780, (**B**) Hey, (**C**) Kuramochi, (**D**) SKOV3 (**E**) TykNu, (**F**) ID8 Trp53−/−. Fold change was calculated relative to the reference gene TBP. A t-test was performed for statistical significance. *p < 0.05 compared to Mock; ^#^p < 0.05 compared to NextA; ^+^p < 0.05 compared to Aza.

We have previously described the immunomodulatory effects of DNMTi and HDAC6i individually^10,40^. We assessed the expression of a panel of interferon stimulated genes (ISGs) *OASL, IFI44L, IFNB1*, and *IFI27* and cytokines *CCL2, CCL5, CXCL10* to determine the immune effects of combination therapy. Both ISGs and cytokines were upregulated after treatment with NextA and Aza in human (A2780, Hey, Kuramochi, SKOV3, and TykNu) and mouse (MOSE ID8 Trp53−/−) ovarian cancer cell lines (Fig. 2). In the A2780, Hey, and ID8 Trp53−/− cell lines, both Aza and NextA significantly increased the expression of cytokines and interferon genes, but the combination significantly increased the expression of every gene tested over the individual treatments. The TykNu cell line saw significant increases with Aza alone more so than with NextA, and combining the treatments only increased expression over Aza alone for two out of seven genes. The Kuramochi cell line exhibited some upregulation with NextA and Aza, and the combination was significantly higher than a single treatment for five out of seven genes. The SKOV3 cell line had the least response to epigenetic therapy, with minimal significant increases in gene expression and only one gene, *OASL*, that was significantly increased with the combination therapy. Thus, while exhibiting some variability between cell lines, the NextA/Aza combination significantly increased cytokine and interferon stimulated gene expression in 5/6 ovarian cancer cell lines tested compared to each individual treatment.

### Combining Nexturastat A and 5-Azacytidine decreases DNMT1 expression

**Figure 3:**
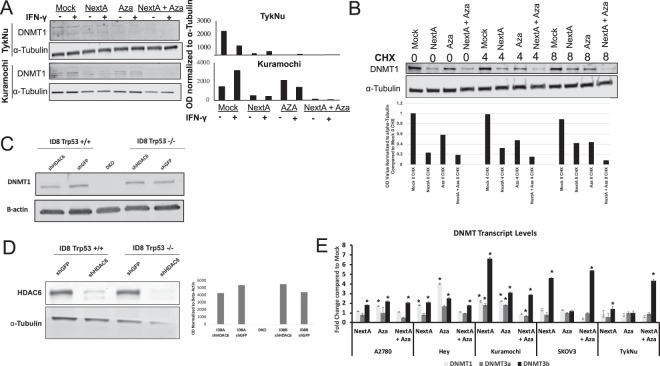
DNMT1 protein levels are decreased by combination treatment of DNMTi and HDAC6i. (**A**) Ovarian cancer cell lines were treated as in Fig. 1 and protein was extracted at Day 7 after treatment with IFN-gamma (IFN-γ+) (to assess MHC I and PD-L1 expression, in later figures) or control (IFN-γ -). Protein was isolated and immunoblots were run for the DNMT1 protein and α-tubulin as a loading control. Immunoblot membranes were cut and probed separately for DNMT1 (about 188 kDa) and α-tubulin (50 kDa). Cropped blots are shown here, and black lines indicate where one part of the blot ends and another begins. Figure S7B shows the entire blot images. (**B**) The TykNu cell line was treated as in (**A**) and the protein synthesis cycloheximide added to cells on Day 7 for 0, 4, and 8 hours at 10 μM as indicated on the blot. Protein was isolated and immunoblots were run for the DNMT1 protein and α-tubulin as a loading control. Immunoblot membranes were cut and probed separately for DNMT1 (about 188 kDa) and α-tubulin (50 kDa). Cropped blots are shown here, and black lines indicate where one part of the blot ends and another begins. Figure S7C shows the entire blot images. (**C**) Stable knockdowns of the HDAC6 protein were generated in the ID8 Trp53+/+ and Trp53−/− cell lines^46^. Protein was extracted and immunoblots were run for the DNMT1 protein with B-actin as a loading control. Immunoblot membranes were probed for DNMT1 (about 188 kDa) and α-tubulin (50 kDa). Cropped blots are shown here and black lines indicate where one part of the blot ends and another begins. Figure S7D shows the entire blot images. (**D**) Immunoblot showing knockdown of HDAC6 protein with a-Tubulin as a loading control. Protein was extracted and immunoblots were run for the HDAC6 protein with B-actin as a loading control. Immunoblots were probed for HDAC6 (131 kDa) and tubulin (50 kDa). Cropped blots are shown here and black lines indicate where one part of the blot ends and another begins. Figure S7E shows the entire blot images. (**E**) Ovarian cancer cell lines were treated as in Fig. 1 and RNA was extracted at Day 7. qRT-PCR was run for DNMT1, DNMT3a, and DNMT3b and TBP was used as a reference gene. *p < 0.05 compared to Mock.

We performed immunoblotting and qRT-PCR of the DNMT1 maintenance methyltransferase to confirm the effectiveness of DNMTi and HDAC6i treatment. Aza degrades the DNMT1 protein, as expected (Fig. 3A). While the mechanism of action of NextA, an HDAC6i and Aza, a DNMTi are well known, we found that the combination of both epigenetic modulators affects DNMT1, with an additive decrease in the protein expression of DNMT1 when combining both agents (Fig. 3A). Interestingly, when we used cycloheximide to inhibit protein synthesis, DNMT1 still showed significantly lower protein levels with the combination treatment (Fig. 3B). This implies that the epigenetic drugs cause degradation of the DNMT1 protein. A stable knockdown of HDAC6 in ID8 Trp53−/− cells did not decrease the DNMT1 protein (Fig. 3C,D) suggesting that the DNMT1 inhibition noted was an off-target effect of NextA rather than an HDAC6-mediated effect. We analyzed transcript levels of the maintenance (*DNMT1*) and de novo (*DNMT3A, DNMT3b*) methyltransferases and observed changes in the transcript levels but no consistent decrease in *DNMT1* RNA levels (Fig. 3E). The more dramatic depletion of DNMT1 by the combination of both HDAC6i and DNMTi may explain why the addition of HDAC6i to DNMTi increases the expression of the immunomodulatory pathways profiled in Fig. 2.

### Combination of Nexturastat A and 5-Azacytidine affects PD-L1 expression

**Figure 4:**
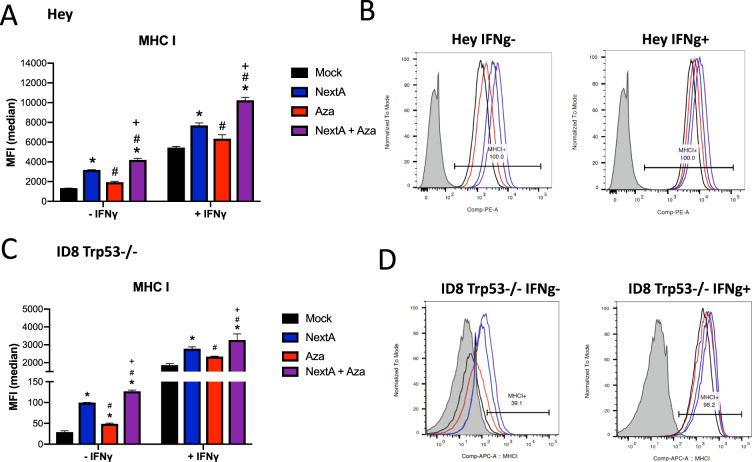
MHC I expression on the cell surface is increased after DNMTi and HDAC6i treatment. Hey human ovarian cancer cells and ID8 Trp53−/− mouse ovarian cancer cells were treated as in Fig. 1 and cells were prepared at Day 7 after treatment with IFN-gamma (IFN-γ+) or control (IFN-γ−). Cells were stained for MHC I surface marker by flow cytometry. (**A**) MHC I MFI in Hey cell line. (**B**) Representative histogram of Hey MHC I expression (**C**) MHC I MFI in ID8 Trp53−/− cell line. (**D**) Representative histogram of ID8 Trp53−/− MHC I expression. A one way ANOVA was performed for statistical significance: *p < 0.05 compared to Mock; ^#^p < 0.05 compared to NextA; ^+^p < 0.05 compared to Aza.

To further assess the downstream effects of the Type I interferon response, we measured the cell surface expression of MHC class I, which presents antigens to T cells, in the ID8 Trp53/- mouse ovarian cancer cell line^46^ and the Hey human ovarian cancer cell line. MHC class I is upregulated in cells treated with NextA and significantly further increased by NextA + Aza treatment in both human (Fig. 4 A, 4B) and mouse (Fig. 4 C, 4D) ovarian cancer cells.

One of the downstream effects of the Type I interferon response is upregulation of the immunosuppressive ligand PD-L1. We found that the combination of Aza and NextA decreased the expression of whole-cell PD-L1 protein more so than each agent alone (Fig. S3A). However, when assessed by flow cytometry, PD-L1 levels on the cell surface were increased by the combination of the two drugs (Fig. S3B–E). Several isoforms of PD-L1 exist, including the membrane bound and soluble PD-L1, which is secreted into the serum. PD-L1 is often upregulated as an ISG in the type I interferon response, so we would expect levels to increase and have observed this previously with Aza treatment^10,11^. While the difference in mechanism of membrane-bound versus soluble PD-L1 has yet to be elucidated, we show here that total protein levels differ significantly from membrane-bound PD-L1 ligand.

### Combination of NextA and Aza in cisplatin-resistant cell lines

We extended our findings in Figs. 1–4 to ovarian cancer cell lines generated to become cisplatin resistant^47^ to mimic human ovarian cancer that does not respond to cisplatin chemotherapy. The IGROV-1 CR and SKOV3 CR cell lines have cisplatin IC50s increased about fivefold that of their parent cell lines (IGROV-1 CR = 1.50 uM, SKOV3 CR = 10.4 uM)^47^. These cell lines exhibited different levels of HDAC1, HDAC2, and HDAC6 proteins (Fig. S4A). Treating SKOV3 CR with NextA, Aza, and NextA + Aza upregulated *CCL2* and *OASL* genes, similar to the SKOV3 parental cell line, with both genes significantly higher in the combination treatment than either NextA or Aza alone (Fig. S4B). The IGROV-1 cell line was much less responsive to epigenetic therapy, with only the cytokine *CCL2* showing a significant increase over the individual therapies (Fig. S4C). These cell lines exhibited similar decrease of total PD-L1 protein (Fig. S4D,F) with the NextA and NextA+ Aza treatment as well as a decrease of the DNMT1 protein with all epigenetic treatments but most pronounced in the combination treatment (Fig. S4D,F). Similar to the other cell lines, they also exhibited an increased in PD-L1 protein expression on the cell surface with epigenetic treatment (Fig. S3D,E). Lastly, they exhibited an increase in MHC I expression with NextA or NextA + Aza treatment, similar to the other cell lines profiled (Fig. S4E,G).

### Combination of Nexturastat A and 5-Azacytidine increases tumor immunity and decreases tumor burden in mice

**Figure 5:**
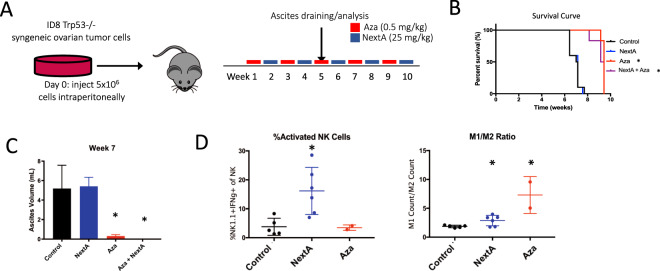
DNMTi+ HDAC6i combination treatment increases survival and changes the tumor microenvironment in the ID8 Trp53−/− mouse model of ovarian cancer. (**A**) Schematic of drug treatment. Mice were given intraperitoneal injections with 5.0 × 10^6^ MOSE ID8 Trp53−/− murine ovarian cancer cells^46^. Treatment began 1 week after tumor inoculation and ended when the tumor burden was deemed excessive for the animal as per our IACUC protocol. Mice were injected with 5-azacytidine (Aza) and Nexturastat (NextA) 5 days a week on alternating weeks. Mice in the 5-azacytidine group were given 0.5 mg/kg of 5-azacytidine dissolved in 100 ul of PBS solution daily. Mice in the NexturastatA group were given 25 mg/kg of Nexturastat A dissolved in 100 ul PEG vehicle solution(70% Polyethylene glycol, 10% Tween80, 20% absolute ethanol) daily. Mice in the NextA+Aza group were given 5-azacytidine and NexturastatA on an alternating weekly basis. Mice in the control group were given a drug vehicle comprised of 70% Polyethylene glycol, 10% Tween 80, and 20% 200 proof absolute ethanol as a vehicle control for Nexturastat or PBS as a vehicle control for 5-azacytidine. (**B**) Both the Aza and NextA + Aza groups exhibited significantly longer survival compared to the Mock and NextA groups (*p < 0.05 by the log-rank (Mantel-Cox) test). (**C**) Ascites volume (tumor burden) was measured at Week 7. *p < 0.05 by one way ANOVA. (**D**) Ascites were isolated from mice in three groups with ascites at Week 7 and immunophenotyping was performed for M1 and M2 macrophages and NK cells. *p < 0.05 by one way ANOVA.

Having observed increased immunogenicity with our combination treatment, we tested whether combining HDAC6i with DNMTi in a mouse model of ovarian cancer could decrease tumor burden and prolong survival. We treated mice bearing ID8 Trp53−/− ovarian cancer^46^ with intraperitoneal injections of NextA, Aza, or Aza+NextA and measured their tumor burden and survival. We collected the ascites fluid from the mice and subjected it to immunophenotyping. Either Aza or the combination of Aza+NextA significantly increased survival in this model, but NextA treatment alone did not (Fig. 5A,B). This survival difference correlated with decreased ascites volume in the Aza and NextA + Aza groups (Fig. 5C). We believe that this effect is due to an increase in immunogenic effects from the combination treatments, with a change in the tumor microenvironment favoring the presence of activated NK cells (mostly driven by NextA) and an increase in M1 macrophages (driven by Aza) (Fig. 5D).

**Figure 6:**
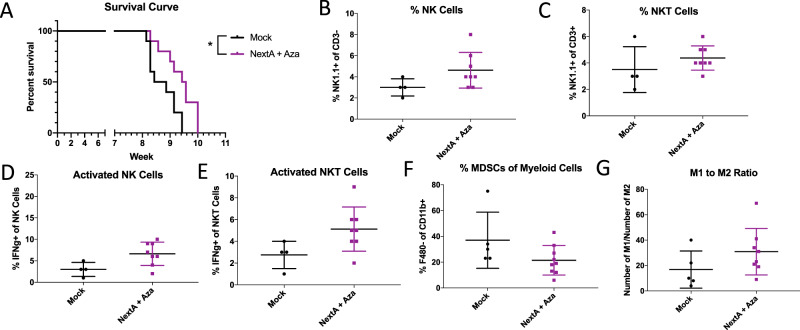
DNMTi+ HDAC6i combination treatment improves response and changes the TME in the ID8 Trp53−/− mouse model of ovarian cancer. (**A**) C57Bl6 mice were injected with ID8 Trp53−/− cells and treated with 0.5 mg/mL Aza daily every other week and 25 mg/kg NextA daily every other week, alternating weeks as in Fig. 5A. The NextA + Aza group had significantly longer survival compared to the Mock group (p = 0.0128 by log-rank (Mantel-Cox) test)). Ascites were isolated at Week 9 and immunophenotyping was performed to measure: (**B**) Natural Killer (NK) cells as % NK of CD3- immune cells, (**C**) Natural Killer T cells as % NK of CD3+ immune cells, (**D**) Activated NK cells (as %IFNg+ of NK cells), (**E)** Activated NKT cells (as %IFNg+ of NK+ CD3+ cells), (**F**) Myeloid Derived Suppressor Cells (MDSCs), and (**G**) ratio of M1 (defined as CD11b+/Ly6C+ Ly6G-/F480+) to M2 (defined as CD11b+/Ly6C-Ly6G+/F480+) macrophages. T-tests were performed to test significance; *p < 0.05. Plots without * indicate non-significant differences.

We performed a second mouse experiment with Mock and Aza + NextA groups and observed a similar significant increase in survival (Fig. 6A). Concomitant with the increased survival, we observed an increase in natural killer cell infiltration into the tumor microenvironment (Fig. 6B,C), and increased activation of NK cells (Fig. 6D,E). We also observed a decrease in the percentage of suppressive myeloid cells (Fig. 6F) and an increase in the M1/M2 macrophage ratio (Fig. 6G) suggesting reversal of the immunosuppressive tumor microenvironment.

**Figure 7:**
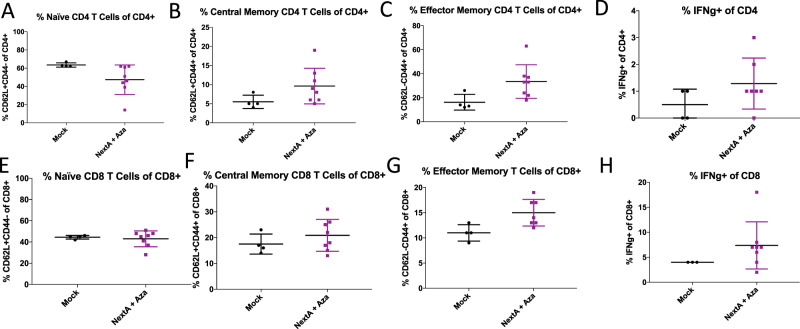
DNMTi+ HDAC6i combination treatment changes the T cell phenotype in the ID8 Trp53−/− mouse model of ovarian cancer. C57Bl6 mice were injected with ID8 Trp53−/− cells and treated with 0.5 mg/mL Aza daily every other week and 25 mg/kg NextA daily every other week, alternating weeks as in Fig. 5A. Ascites was isolated from mice in each group and immunophenotyping was performed to measure: (**A**) Naïve CD4 T cells (defined as %CD62L+CD44- of CD4 cells) (**B**) Central Memory CD4 T cells (defined as %CD62L+ CD44+ of CD4), (**C**) Effector Memory CD4 T cells (defined as CD62L-CD44+ of CD4), (**D)** Activated CD4 T cells (defined as % IFNg+ of CD4 T cells), (**E**) Naïve CD8 T cells (defined as %CD62L+ CD44- of CD8), (**F**) Central Memory CD8 T cells (defined as %CD62L+ CD44+ of CD8), (**G**) Effector Memory CD8 T cells (defined as CD62L-CD44+ of CD8), and (**H**) Activated CD8 T cells (defined as % IFNg+ of CD8 T cells). T-tests were performed to test significance; *p < 0.05. Plots without * indicate non-significant differences.

Interestingly, the combination treatment seems to promote the Effector and Central Memory phenotype of CD4 T cells. We observed an increase in Effector Memory T cells (T_EM_), both CD4 and CD8 (Fig. 7C,G) and Central Memory T cells (T_CM_), both CD4 and CD8 (Fig. 7B,F), and a concomitant decrease in Naïve CD4 and CD8 T cells (Fig. 7A,E). We observed increased activation of CD4 (Fig. 7D) and CD8 (Fig. 7H) cells, as measured by interferon-gamma staining. Thus, the combination of both therapies decreases immunosuppressive cells in the tumor microenvironment and increases recruitment and activation of tumor-killing immune cells. None of these changes were significant compared to the Mock treatment as there was considerable variability within groups, but the epigenetic therapy combination exhibits a trend towards a more immunogenic tumor microenvironment.

**Figure 8:**
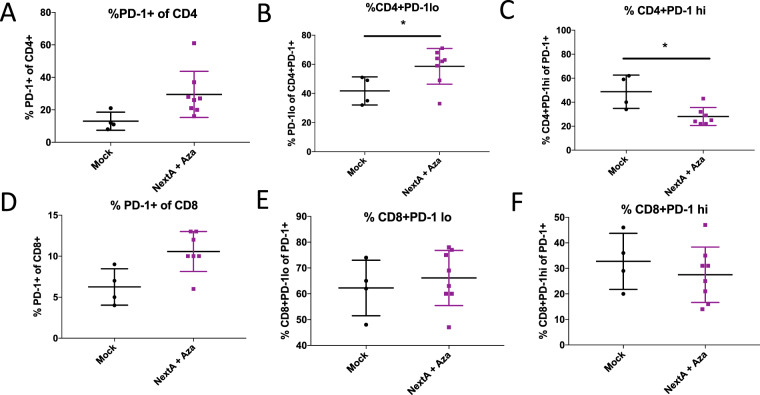
DNMTi+ HDAC6i combination treatment affects PD-1 on T cells in the ID8 Trp53−/− mouse model of ovarian cancer. C57Bl6 mice were injected with ID8 Trp53−/− cells and treated with 0.5 mg/mL Aza daily every other week and 25 mg/kg NextA daily every other week, alternating weeks as in Fig. 5A. Ascites was isolated from mice in each group and immunophenotyping was performed to measure: (**A**) PD-1+ cells of CD4, (**B**) PD-1lo cells of CD4, (**C**) PD-1hi cells of CD4, (**D**) PD-1+ cells of CD8, (**E**) PD-1lo cells of CD8, and (**F**) PD-1hi cells of CD8. T-tests were performed to test significance; *p < 0.05. Plots without * indicate non-significant differences.

We also noted an increase in PD-1+ CD4 and CD8 T cells (Fig. 8). Because of this and the increase in PD-L1 (Fig. S3), we hypothesized that addition of anti-PD-1 would increase survival in this mouse model. However, neither anti-PD-1 alone nor the combination of anti-PD-1 + Aza + NextA showed significantly improved survival compared to Aza + NextA treatment (Figs S4, S5). With Aza + NextA treatment we also observed a significant increase in PD-1^lo^ CD4 T cells (Fig. 8B) and concomitant significant decrease in PD-1^hi^ CD4 T cells (Fig. 8E), suggesting increased levels of activation and decreased levels of exhaustion respectively.

## Discussion

In this study, we demonstrate that combining the epigenetic modifiers HDAC6i (NextA) and DNMTi (Aza) upregulates the immune response in ovarian cancer in both *in vivo* and *in vitro* models. We found that the combination of NextA with Aza upregulates the type I interferon response significantly more than either treatment alone. We observed upregulation of interferon-stimulated genes such as *OASL, IFI44L, IFNB1* and an increase in the expression of cytokines (including *CCL2, CCL5*, and *CXCL10*) (Fig. 2). Based on our previous work^12^, we expect this type I interferon response to recruit and activate host immune cells.

Downstream of the interferon response, both the MHC class I antigen-presenting complex (Fig. 4) and the immunosuppressive PD-L1 ligand were increased on the cell surface by Aza+ NextA treatment (Fig. S3). The significant increase of MHC I on the cell surface promotes better antigen presentation to T cells^48^. PD-L1 is an interferon stimulated gene, increased by the type I interferon response, and we have previously shown that Aza increases PD-L1 expression^10,11^. The PD-L1 results are complex, as total PD-L1 protein was decreased after treatment with NextA and Aza (Fig. S3). Several isoforms of PD-L1 exist, including the membrane bound and soluble PD-L1, which is secreted into the serum^49^. While the difference in mechanism of membrane-bound versus soluble PD-L1 has yet to be elucidated, we show here that total protein levels differ significantly from membrane-bound PD-L1 ligand. Others have shown that cisplatin-resistant cell lines have slightly higher PD-L1 surface expression than their parental cell lines^50^. We found that the Hey (cisplatin sensitive) and SKOV3 CR (cisplatin resistant) had the highest levels of PD-L1 by MFI (Fig. S3) while the ID8 Trp53−/− (cisplatin sensitive) and IGROV-1 CR (cisplatin resistant) had lower levels of PD-L1. Thus we did not observe a correlation with cisplatin status. All ovarian cancer cell lines tested demonstrated a significant induction of PD-L1 by interferon gamma, which was further increased by epigenetic therapy that activated type I interferon signaling (Fig. S3).

In the ID8 Trp53−/− mouse model of ovarian cancer, we found that combination Aza/NextA treatment recruits immune cells, specifically CD8 and NKT cells that can kill tumor cells (Figs. 5–8). We observed a decrease in immunosuppressive myeloid cells and an increase in the M1/2 macrophage ratio, promoting a more immunogenic tumor microenvironment. Interestingly, we also observed differences in the naïve/effector/central memory T cell populations, with the Aza/NextA treatment increasing the percentage of the Effector Memory T cells and Central Memory T cells within the tumor microenvironment. These data suggest that Aza/NextA treatment either increases T cell trafficking to the tumor microenvironment from secondary lymphoid organs or that it promotes the development of Central Memory and Effector Memory T cells (Figs. 6–8). DNA methylation is known to control aspects of T cell differentiation^51–53^ and exhaustion^54,55^ and DNMTi can significantly effect T cell phenotype.

The observed alteration of the tumor microenvironment by HDAC6i and DNMTi contributed to a modest increase in survival in our murine ovarian cancer model. We noted a decrease in tumor burden (measured by ascites volume) and a significant increase in survival in the mice treated with Aza and Aza/NextA (Figs. 5 and 6). It is important to note that while PD-1 expression was upregulated in the mouse model, anti-PD-1 did not confer better survival rates in the murine ovarian cancer model either alone or in combination with HDAC6i+ DNMTi. This is in contradiction to previous work, which has shown a synergistic effect in combining NextA with anti-PD-1 in melanoma^37,40^. This may occur because of the significant increase in PD-L1 cell surface protein caused by NextA+Aza, downstream of the interferon response (Fig. S3D).

We conclude that combining DNMTi and HDAC6i in ovarian cancer cell lines increases immunogenicity as measured by type I interferon signaling, cytokine production, and MHC I expression on the cell surface significantly increased over either treatment alone. However, the combination of these epigenetic modifiers in the ID8 MOSE Trp53−/− model of ovarian cancer produced only modest effects on survival, although it did promote a more immunogenic tumor microenvironment. We observed increased PD-L1 on tumor cells and increased PD-1 on T cells with DNMTi + HDAC6i and thus tested combination treatment with anti-PD-1, but this produced no benefit, perhaps because of the very high levels of PD-L1 ligand produced by the combination treatment. We thus anticipate future work combining DNMTi and HDAC6i with immunotherapies besides checkpoint blockade, such as antigen-specific T cells or cancer vaccines, to promote anti-tumor immunity to eradicate ovarian cancer cells.

## Methods

### Cell Culture

The following cell lines were a kind gift from Dr. Steve Baylin’s laboratory (Johns Hopkins University): A2780, Hey, Kuramochi, TykNu, SKOV3. The following cell lines were provided by Wenge Zhu’s laboratory at George Washington University: IGROV-1 CR, SKOV3 CR. The following cell lines were a kind gift from Dr. Iain McNeish’s laboratory (Imperial College London)^46^: ID8 wild type, ID8 Trp53^−/−^. Cell lines were identity tested every six months using a short tandem repeat (STR) profiling service (Johns Hopkins Genetic Resources Core Facility) and tested for mycoplasma contamination every three months. STR profiling by the GRCF is carried out following the ANSI/ATCC ASN-0002-2011, Authentication of Human Cell Lines: Standardization of STR Profiling. For cell lines derived from repositories, the profile of the cell line is used for comparison using verification tools found on the repository web sites to determine relatedness of the line to those held by the repositories. GRCF uses the following databases to generate the reports: Leibniz Institute DSMZ German Collection of Microorganisms and Cell Cultures, American Tissue Culture Collection, Japanese Collection of Research Bioresources. The algorithms used in the American National Standards Institute (ANSI) and the American Type Culture Collection Standards Development Organization’s (ATCC SDO) report ANSI/ATCC ASN-0002-2011 are used to calculate percent match or evaluation value (EV) which are then reported to our lab.

The Hey, TykNu, SKOV3, and A2780 cell lines were cultured in RPMI 1640 (Corning, 10-104-CV) with 10% Fetal Bovine Serum (X&Y Cell Culture, FBS-500-HI), and 1% penicillin and streptomycin solution (Gibco, 15070063). The Kuramochi cell line was cultured in RPMI 1640 media (Corning, 10-104-CV) with 10% fetal bovine serum (X&Y Cell Culture, FBS-500-HI), 1% penicillin and streptomycin solution (Gibco, 15070063) and 1% non-essential amino acids (Gibco, 11140050). The OVCAR3 cell line was cultured in RPMI media with 20% fetal bovine serum (X&Y Cell Culture, FBS-500-HI), 1% penicillin streptomycin solution (Gibco, 15070063), and 1% bovine insulin (I0516-5ML). The ID8 murine cell line was cultured in DMEM media (Gibco, 10569044) with 4% fetal bovine serum (X&Y Cell Culture, FBS-500-HI), 1% penicillin streptomycin solution (Gibco, 15070063), 5 ugμ/mL insulin, 5 bug/mL transferrin, and 5 ug/mL sodium selenite (Gibco, 41400045).

### Drug treatments

Cells were cultured in T75 dishes (Greiner, 658170) to form a monolayer at 80% confluency. Combination cells were treated with 500 nM 5-azacytidine (Sigma-Aldrich) once a day for 3 days or PBS once a day for 3 days. Cells were passaged on the fourth day and split into 2 flasks to be treated with Nexturastat (StarWise, LLC) or DMSO for vehicle control at the desired dose based on the cell line for the following 3 days. On the first day of Nexturastat treatment, one flask from each treatment group was stimulated with 250 ng/mL IFNγ (Peprotech, 300-02). For isogeneic ID8 murine cell lines, on the first day of HDACi treatment cell were treated with 250 ng/mL IFNγ (Peprotech, 315-05). On the seventh day, cell pellets were collected for Western Blot or flow cytometry analysis. For cycloheximide experiments, cells were treated on the seventh day of the experiment for 0, 4 or 8 hours with 10 μM cycloheximide (Sigma, C4859).

### Western blot

Isolated cell pellets were lysed in RIPA buffer (Pierce, 89900) with 1X protease and phosphatase inhibitor (Pierce, A32961). Lysates were sonicated in a Bioruptor™ (Diagenode, Denville, NJ, USA) on ice for 8 minutes (8 cycles of 30 s on, 30 s off). Protein concentration was determined using a Pierce BCA Protein Assay Kit (Thermo Fisher Scientific, 23225) according to the manufacturer’s protocol. Samples were mixed with NuPAGE LDS 4x loading gel (NP0007) and NuPAGE 10x reducing agent (NP0009), and boiled at 95 °C. Next, samples were loaded onto 4–20% (BioRad, 4561093) or 10% gels (BioRad, 4561033) and transferred to LF PVDF (BioRad, 170–4274). Membranes were blocked with LI-COR Biosciences (Lincoln, Nebraska, USA) Odyssey Blocking Buffer (927–40100) or 5% milk-PBS-Tween. Bands were detected using Azure Biosystems (Dublin, California, USA) Imaging System c600. The antibodies used for immunoblotting included: DNMT1 (Sigma, D4692), PD-L1 (ProSci, 4059), HDAC1 (Cell Signaling, 2062), HDAC2 (Cell Signaling, 2540), HDAC6 (Assay Biotech, C0026), acetyl-alpha Tubulin (Cell Signaling, 3971), alpha-Tubulin (Cell Signaling, 3873).

### RNA extraction and analysis

Cells were cultured in T75 dishes to form a monolayer at 80% confluency. Combination cells were treated with 500 nM 5-azacytidine or PBS for 3 days. Cells were passaged on the fourth day and split into 2 flasks to be treated with Nexturastat or vehicle at the desired dose based on the cell line for the following 3 days. On the last day of HDACi treatment, one flask from each treatment group was stimulated with 250 ng/mL IFNγ (Peprotech, 300-02) for 4 hours. For isogeneic ID8 murine cell lines, cell were stimulated with 250 ng/mL If (Peprotech, 315-05) for 4 hours. On the seventh day cell pellets were collected for qPCR analysis.

Total RNA was extracted using the TRIzol method (Life Technologies). RNA concentration was determined using the Thermo Scientific NanoDrop One machine and software (Thermo Scientific, 840274100PR2). One microgram total RNA was DNAse I (Thermo Scientific) treated and used to generate cDNA with the Applied Biosystems High-Capacity cDNA Reverse Transcriptase Kit (Applied Biosystems, 4368814). All RT-qPCRs were run on the Applied Biosystems Quantstudio3 Real-Time PCR System, 96-well plate reader (Applied Biosystems, A28137) and using TaqMan assay Gene Expression Array (Thermo Fisher Scientific, 4331182) in human for *OASL, IFI44L, IFNb1, CXCL10, CCL2, CCL5, IFI27, CD247, DNMT1, DNMT3a, DNMT3b* and in mouse (Thermo Fisher Scientific, 4331182) for *OASL, IFI44L, IFNb1, CXCL10, CCL2, CCL5, IFI27, and CD247*. TBP was used as a housekeeping gene for both human and murine cell lines. The ΔΔCt method was used to calculate relative expression levels. Reverse transcriptase negative cDNA synthesis reaction was performed on at least one sample per 96-well plate along with a water control to ensure accurate results.

### Flow cytometry

Ascites and murine spleens were collected from 5 to 10 mice per group and incubated in ACK buffer (Thermo Fisher Scientific) to lyse red blood cells for 10 min and then washed. Ascites from each mouse were individually prepared for flow cytometry. The single cells collected were cultured for 4 hours in RPMI with 10% FBS and in the presence of 2 ul/ml Cell Stimulation Cocktail plus protein transport inhibitors (eBioscience, 00497503). Cells were then washed and stained for the T cell panel including: Live/Dead (Thermo Fisher Scientific, L34965), CD45 (Biolegend, 103132), CD3 (Biolegend, 152316), CD4 (Biolegend, 100453), CD8 (Biolegend, 100766), NK1.1 (Biolegend, 108753) CD 279 (Biolegend, 135205). After incubation, the cells were permeabilized (FoxP3 staining buffers; BD Bioscience, 563486). Intracellular staining was performed for FoxP3 (Biolegend, 505806) and IFN-γ (BD Bioscience, 563486).

A proportion of these same ascites cells were also washed and stained for the myeloid panel including: Live/Dead(Thermo Fisher Scientific, L34965), CD45 (Biolegend, 103132), Ly6C (Biolegend, 128046), Ly6G (Biolegend, 127639), CD11b (Biolegend, 101243), F4/80 (Biolegend, 123110), I-A/I-E (Biolegend, 107605), CD80 (Biolegend, 104732), CD206 (Biolegend, 141708), CD40 (Biolegend, 124622), CD3 (Biolegend, 100216), B220 (Biolegend, 103232), and NK1.1 (Biolegend, 108730).

Murine ovarian cancer cell lines from day 7 of the protein protocol were stained for PD-L1/CD274 (BD Bioscience, 558091), PD-L2/CD273 (Biolegend, 107214), MHC-I (Invitrogen, 17-5958-82), and MHC-II (Invitrogen 56-5321-82).

Human ovarian cancer cells from day 7 of the protein protocol were stained for CD274 (eBioscience,12-5983-42) and MHC-I (eBioscience, 12-9983-42).

All cells were fixed after staining using Foxp3/Transcription Factor Staining Buffer Set (eBioscience, 00-5523-00) and run on a 3-laser, 12-color BD Celesta analyzer. All flow cytometry data were analyzed using the FlowJo Software 10.6 and statistical outliers were removed using Pierce’s criterion.

### Cellular toxicity assay

Drug toxicity levels were measured using the CellTox Assay (Promega, G8741). Cells were seeded into a 96-well dish and treated with 500 nm 5-azacytidine and varying levels of HDACi to determine suitable dosage. Cells were treated with fluorescent dye and incubated for 4 days measuring cell death according to the manufacturer’s protocol. For longer treatments, cells were seeded in a 96-well place and treated with 500 nm 5-azacytidine for 4 days and then varying levels of HDACi. Cells were treated with a fluorescent dye on the fourth day and incubated to measure cell death. Assay measurements were collected using SpectraMax i3x multi-mode microplate reader (Molecular Devices, USA) at the recommended wavelength. For our analysis, TritonX (Sigma, 9002-93-1) was used as the positive control compound to simulate cell death on all Petromax read days. Results were analyzed according to manufacture recommendations.

### HDAC enzymatic inhibition assay

HDACi enzymatic function was measured using HDACglo class I/IIa Assay (Promega, G6420). Cells were seeded in a 96-well dish and treated with 500 nM 5-azacytidine and varying levels of HDACi to determine appropriate HDACi dosage. Assay measurements were collected using SpectraMax i3x multi-mode microplate reader (Molecular Devices, USA) at the recommended wavelength. Triclosan A, Givinostat, and Panobinostat (SelleckChem) were used to compare enzymatic inhibition levels of Nexturastat in each cell line. Results were analyzed according to manufacturer recommendations.

### Mouse experiments

Experiments involving mice were performed in accordance with the approved protocol by the Institutional Care and Use Committee (IACUC) at The George Washington University (Protocol A406). C57B/6 mice were obtained from the Charles River Laboratories (Wilmington, Massachusetts, USA). For *in vivo* tumor studies, all mice were given intraperitoneal injections with 5.0 ×10^6 p53−/− isogeneic murine ovarian cancer cells suspended in 500 uL 1x phosphate buffered saline (PBS) (Corning 21-040-CV). Treatment began 1 week after tumor inoculation and ended when the tumor burden was deemed excessive for the animal. Mice were injected with 5-azacytidine and Nexturastat 5 days a week in alternating treatment (see treatment schemas in Figs. 5 and 6). All mice were given intraperitoneal injections of epigenetic drugs or their appropriate controls. Mice in the 5-azacytidine group were given 0.5 mg/kg of 5-azacytidine dissolved in 100 ul of PBS solution daily. Mice in the NexturastatA group were given 25 mg/kg of Nexturastat A dissolved in 100 ul PEG vehicle solution(70% Polyethylene glycol, 10% Tween80, 20% absolute ethanol) daily. Mice in the NextA+Aza group were given 5-azacytidine and NexturastatA on an alternating weekly basis. Mice in the control group were given a drug vehicle comprised of 70% Polyethylene glycol (Sigma 764752), 10% Tween 80 (Sigma P5188), and 20% 200 proof absolute ethanol as per the manufacturer guidelines as a vehicle control for Nexturastat or PBS as a vehicle control for 5-azacytidine.

Tumor burden was assessed via measurement of body weight, amount of ascites drained from the mice at the point where they had gained 20 to 30% of their body weight, or abdominal circumference measurements. Statistical outliers were removed using Pierce’s criterion. While each experimental group started with 10 mice, at the time ascites were drained some mice had died, and other treated mice did not have ascites yet. Furthermore, some mice only yielded enough cells to run one panel for flow cytometry, which led to decreases in mouse numbers and differences between groups in some figures.

### Statistical analysis

All experiments were done in triplicate unless otherwise noted. Analysis completed using unpaired t-tests and ANOVA with significance at p <  0.05, and the Kaplan-Meier survival curves were conducted using GraphPad Prism 7. All analyses of cell toxicity, enzyme inhibition, and RT-qPCR were conducted using Microsoft Excel Software (Microsoft, Redmond, WA).

## Supplementary information

Supplemental Information.
