# Author Correction: Combining DNMT and HDAC6 inhibitors increases anti-tumor immune signaling and decreases tumor burden in ovarian cancer

**DOI:** 10.1038/s41598-021-04066-1

**Published:** 2021-12-20

**Authors:** Sara Moufarrij, Aneil Srivastava, Stephanie Gomez, Melissa Hadley, Erica Palmer, Paul Tran Austin, Sarah Chisholm, Noor Diab, Kyle Roche, Angela Yu, Jing Li, Wenge Zhu, Micael Lopez-Acevedo, Alejandro Villagra, Katherine B. Chiappinelli

**Affiliations:** 1 The George Washington University Cancer Center, The George Washington University, Washington, DC USA; 2 The Department of Obstetrics & Gynecology, The George Washington University, Washington, DC USA; 3 The Department of Microbiology, Immunology, & Tropical Medicine, The George Washington University, Washington, DC USA; 4 The Department of Biochemistry and Molecular Medicine, The George Washington University, Washington, DC USA; 5 The Institute for Biomedical Sciences, The George Washington University, Washington, DC 20052 USA

Correction to: *Scientific reports* 10.1038/s41598-020-60409-4, published online 26 February 2020

Noor Diab was omitted from the author list in the original version of this Article.

The Author Contributions section now reads:

S.M., A.S., M.L.-A., A.V., and K.B.C. designed the experiments. S.M., A.S., S.G., M.H., P.T.A., S.C., E.P., K.R., N.D., and A.Y. performed experiments. J.L. and W.Z. provided cisplatin-resistant cell lines IGROV-1 CR and SKOV3 CR. S.M., A.S., S.G., A.V., and K.B.C. wrote, edited, and revised the manuscript.

The original Article and accompanying Supplementary Information file has been corrected.

