# Supplementary material for: Combining DNMT and HDAC6 inhibitors increases anti-tumor immune signaling and decreases tumor burden in ovarian cancer
Source: Sci Rep. 2020 Feb 26;10:3470. doi: 10.1038/s41598-020-60409-4 (PMC7044433; doi:10.1038/s41598-020-60409-4)
Supplement: Supplementary file 1 — Supplemental Information. [file 41598_2020_60409_MOESM1_ESM.pdf]

Combining DNMT and HDAC6 inhibitors increases anti-tumor immune signaling and decreases tumor burden in ovarian cancer

Sara Moufarrij[1,2]*, Aneil Srivastava[1,3]*, Stephanie Gomez[1,3], Melissa Hadley[1,3], Erica Palmer[1,4], Paul Tran Austin[1,3], Sarah Chisholm[1,3], Noor Diab[1], Kyle Roche[1,3], Angela Yu[1,3], Jing Li[1,4], Wenge Zhu[1,4], Micael Lopez-Acevedo[1,2], Alejandro Villagra[1,4]#, Katherine B. Chiappinelli[1,3]#

*These authors contributed equally to the work

#Co-corresponding authors

1. The George Washington University Cancer Center
2. The Department of Obstetrics & Gynecology
3. The Department of Microbiology, Immunology, & Tropical Medicine
4. The Department of Biochemistry and Molecular Medicine

The George Washington University, Washington, DC

Supplementary Information File

Figure S1. The HDAC6 inhibitor Nexturastat A causes cell death in ovarian cancer cell lines with and without the DNMTi 5-azacytidine. Ovarian cancer cell lines were treated with indicated concentrations of the HDAC6 inhibitor Nexturastat A (NextA) and 500 nM of the DNMT inhibitor 5-azacytidine (Aza) in the following treatment schema: 3 days of Aza treatment followed by 2 days of NextA treatment. Cytotoxicity was assessed at Days 4 (**A**) and 7 (**B**). Cytotoxicity assays were performed on all cell lines to find an appropriate treatment concentration and to measure the effect of the combination treatment at different time points.

Figure S2. The HDAC6 inhibitor Nexturastat A inhibits HDAC6 in ovarian cancer cell lines. Ovarian cancer cell lines were treated with indicated concentrations of NextA and broader spectrum HDAC inhibitors Givinostat (Class I & Class II HDACi), LBH (pan-HDACi), TSA (pan-HDACi). The percent inhibition of HDAC activity was calculated using the HDAC-Glo assay in the following cell lines: **A)** A2780, **B)** Hey, **C)** Kuramochi, **D)** SKOV3 **E)** TYKnu, **F)** ID8 Trp53-/-. The optimal NextA dose for each cell line was chosen as the dose that inhibited greater than 50% of HDAC activity while not causing more than 20% cytotoxicity.

Figure S3. PD-L1 levels are affected by DNMTi and HDAC6i treatment. A) Ovarian cancer cell lines Hey and Kuramochi were treated as in Figure 1 and protein was extracted at Day 7 after treatment with IFN-gamma (IFN-γ +) or control (IFN-γ -). Protein was isolated and immunoblots were run for PD-L1, acetylated tubulin, and α-tubulin as a loading control. Cropped blots are shown here and black lines indicate where one part of the blot ends and another begins. Figure S7F shows the entire blot images. **B)** Hey human ovarian cancer cells were treated as in Figure 1 and cells were prepared at Day 7 after treatment with IFN-gamma (IFN-γ +) or control (IFN-γ -). Cells were stained for PD-L1 surface marker by flow cytometry. A one way ANOVA was performed for statistical significance: * $p < 0.05$ compared to Mock; # $p < 0.05$

compared to NextA; + $p < 0.05$ compared to Aza. **C)** ID8 Trp53-/- ovarian cancer cells were treated as in Figure 1 and cells were prepared at Day 7 after treatment with IFN-gamma (IFN-γ +) or control (IFN-γ -). Cells were stained for PD-L1 surface marker by flow cytometry. A one way ANOVA was performed for statistical significance: * $p < 0.05$ compared to Mock; # $p < 0.05$ compared to NextA; + $p < 0.05$ compared to Aza. **D)** SKOV3 CR human ovarian cancer cells were treated as in Figure 1 and cells were prepared at Day 7 after treatment with IFN-gamma (IFN-γ +) or control (IFN-γ -). Cells were stained for PD-L1 surface marker by flow cytometry. **E)** IGROV-1 CR human ovarian cancer cells were treated as in Figure 1 and cells were prepared at Day 7 after treatment with IFN-gamma (IFN-γ +) or control (IFN-γ -). Cells were stained for PD-L1 surface marker by flow cytometry.

Figure S4. Epigenetic therapies increase immune signaling in cisplatin-resistant cell lines. A) Protein was isolated from the IGROV-1 CR and SKOV3 CR human ovarian cancer cell lines and levels of HDACs 1, 2, and 6 were assessed. α-tubulin was used as a loading control. HDAC1 and HDAC6 were run on the same blot which was cut to image, then stripped and reprobed for HDAC2 and stripped and reprobed for α-tubulin. Cropped blots are shown here and black lines indicate where one part of the blot ends and another begins. Figure S7G shows the entire blot images. RNA was isolated from the SKOV3 CR **(B)** and IGROV-1 CR **(C)** cell lines at Day 7 of the treatment schema in Figure 1 and qRT-PCR was performed for ISGs *IFI27, OASL, IFI44L*, and *IFNB1* and cytokines *CCL2, CCL5, and CXCL10*. Fold change is indicated relative to Mock for each of the following treatments: NextA (blue), Aza (red), and NextA + Aza (purple). Fold change was calculated relative to the reference gene TBP. A t-test was performed for statistical significance. * $p < 0.05$ compared to Mock; # $p < 0.05$ compared to NextA; + $p < 0.05$ compared to Aza.

The SKOV3 CR cell line were treated as in Figure 1 and protein was extracted at Day 7 after treatment with IFN-gamma (IFN-γ +) (to assess MHC I and PD-L1 expression) or control (IFN-γ -). **D)** Protein was isolated and immunoblots were run for DNMT1, PD-L1, Acetyl-tubulin (AC tubulin), and α-tubulin as a loading control. Cropped blots are shown here, and black lines indicate where one part of the blot ends and another begins. Figure S7H shows the entire blot images. **E)** Cells were prepared at Day 7 after treatment with IFN-gamma (IFN-γ +) or control (IFN-γ -). Cells were stained for MHC I surface marker by flow cytometry. The IGROV-1 CR cell line were treated as in Figure 1 and protein was extracted at Day 7 after treatment with IFN-gamma (IFN-γ +) (to assess MHC I and PD-L1 expression) or control (IFN-γ -). **F)** Protein was isolated and immunoblots were run for DNMT1, PD-L1, Acetyl-tubulin (AC tubulin), and α-tubulin as a loading control. Cropped blots are shown here, and black lines indicate where one part of the blot ends and another begins. Figure S7H shows the entire blot images. **G)** Cells were prepared at Day 7 after treatment with IFN-gamma (IFN-γ +) or control (IFN-γ -). Cells were stained for MHC I surface marker by flow cytometry.

Figure S5. Adding anti-PD-1 treatment to the DNMTi/HDAC6i combination does not increase the epigenetic treatment's effect on tumor burden or survival. A) C57Bl6 mice were injected with ID8 Trp53-/- cells and treated with 0.5 mg/mL Aza daily every other week and 25 mg/kg NextA daily every other week (alternating, see schematic in Figure S5C). Mice were given 200 μg anti-PD-1 or isotype control twice a week. The NextA + Aza and the NextA + Aza + anti-PD-1 groups had significantly longer survival (days indicated next to the legend) compared to the Mock group. * $p < 0.05$ by log-rank (Mantel-Cox) test. **B)** Ascites volume (tumor burden) was measured at Week 8. **C)** Schematic.

Figure S6. Adding anti-PD-1 treatment to the DNMTi/HDAC6i combination does not increase the Combo treatments effect on the immune microenvironment. C57Bl6 mice were injected with ID8 Trp53-/- cells and treated with 0.5 mg/mL Aza daily every other week and 25 mg/kg NextA daily every other week (alternating, see schematic in Figure S5C). Mice were given 200 μg anti-PD-1 or isotype control twice a week. Ascites were drained from the mice and immunophenotyping was performed on all mice as in Figures 6-8.

Figure S7. Full blot images from all immunoblots.

A) Full blot images from **Figure 1A**. Protein was isolated from six different human ovarian cancer cell lines and levels of HDACs 1, 2, and 6 were assessed. α-tubulin was used as a loading control. HDAC1 and HDAC6 were run on the same blot which was cut to image, then stripped and reprobed for HDAC2 and stripped and reprobed for α-tubulin as shown in **Figure 1A**.

B) Full blot images from **Figure 3A**. Ovarian cancer cell lines were treated as in Figure 1 and protein was extracted at Day 7 after treatment with IFN-gamma (IFN-γ +) (to assess MHC I and PD-L1 expression, in later figures) or control (IFN-γ -). Protein was isolated and immunoblots were run for the DNMT1 protein and α-tubulin as a loading control. Immunoblot membranes were cut and probed separately for DNMT1 (about 188 kDa) and α-tubulin (50 kDa).

C) Full blot images from **Figure 3B.** The TykNu cell line was treated as in A) and the protein synthesis cycloheximide added to cells on Day 7 for 0, 4, and 8 hours at 10 μM as indicated on the blot. Protein was isolated and immunoblots were run for the DNMT1 protein and α-tubulin as a loading control. Immunoblot membranes were cut and probed separately for DNMT1 (about 188 kDa) and α-tubulin (50 kDa). Cropped blots are shown in **Figure 3B**.

D) Full blot images from **Figure 3C.** Stable knockdowns of the HDAC6 protein were generated in the ID8 Trp53 +/+ and Trp53 -/- cell lines (Bitler et al., 2015). Protein was extracted and

immunoblots were run for the DNMT1 protein with B-actin as a loading control. Immunoblot membranes were probed for DNMT1 (about 188 kDa) and α-tubulin (50 kDa). Cropped blots are shown in **Figure 3C**.

E) Full blot images from **Figure 3D**. Immunoblot showing knockdown of HDAC6 protein with a-Tubulin as a loading control. Protein was extracted and immunoblots were run for the HDAC6 protein with B-actin as a loading control. Immunoblots were probed for HDAC6 (131 kDa) and tubulin (50 kDa). Cropped blots are shown in **Figure 3D.**

F) Full blot images from **Figure S3A**. TykNu and Kuromochi lysates were made and immunoblots were run for PD-L1 (40 kDa), acetylated tubulin (52 kDa), and α-tubulin (50 kDa) as a loading control. Cropped blots are shown in **Figure S3A**.

G) Full blot images from **Figure S4A**. Protein was isolated from the IGROV-1 CR and SKOV3 CR human ovarian cancer cell lines and levels of HDACs 1, 2, and 6 were assessed. α-tubulin was used as a loading control. HDAC1 and HDAC6 were run on the same blot which was cut to image, then stripped and reprobed for HDAC2 and stripped and reprobed for α-tubulin. Cropped blots are shown in **Figure S4A**.

H) Full blot images from **Figure S4D, S4F**. The SKOV3 CR and IGROV-1 CR cell lines were treated as in Figure 1 and protein was extracted at Day 7 after treatment with IFN-gamma (IFN-γ +) (to assess MHC I and PD-L1 expression) or control (IFN-γ -). Protein was isolated and immunoblots were run for DNMT1, PD-L1, Acetyl-tubulin (AC tubulin), and α-tubulin as a loading control. Cropped blots are shown in **Figure S4D, S4F**.

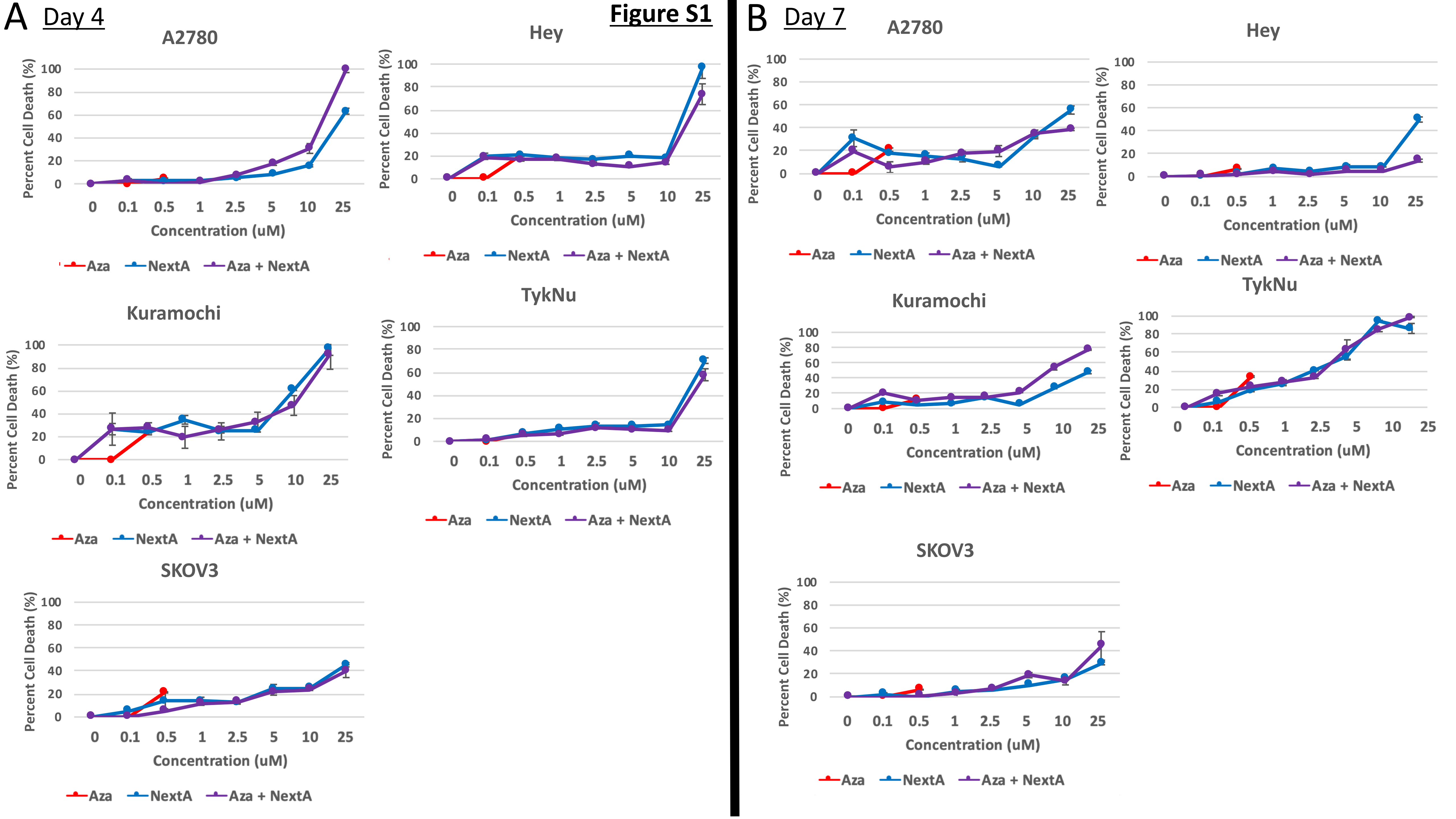

Figure S1
A
Day 4
A2780
Hey
Kuramochi
TykNu
SKOV3
B
Day 7
A2780
Hey
Kuramochi
TykNu
SKOV3
Percent Cell Death (%)
Concentration (uM)
0
0.1
0.5
1
2.5
5
10
25
Aza
NextA
Aza + NextA

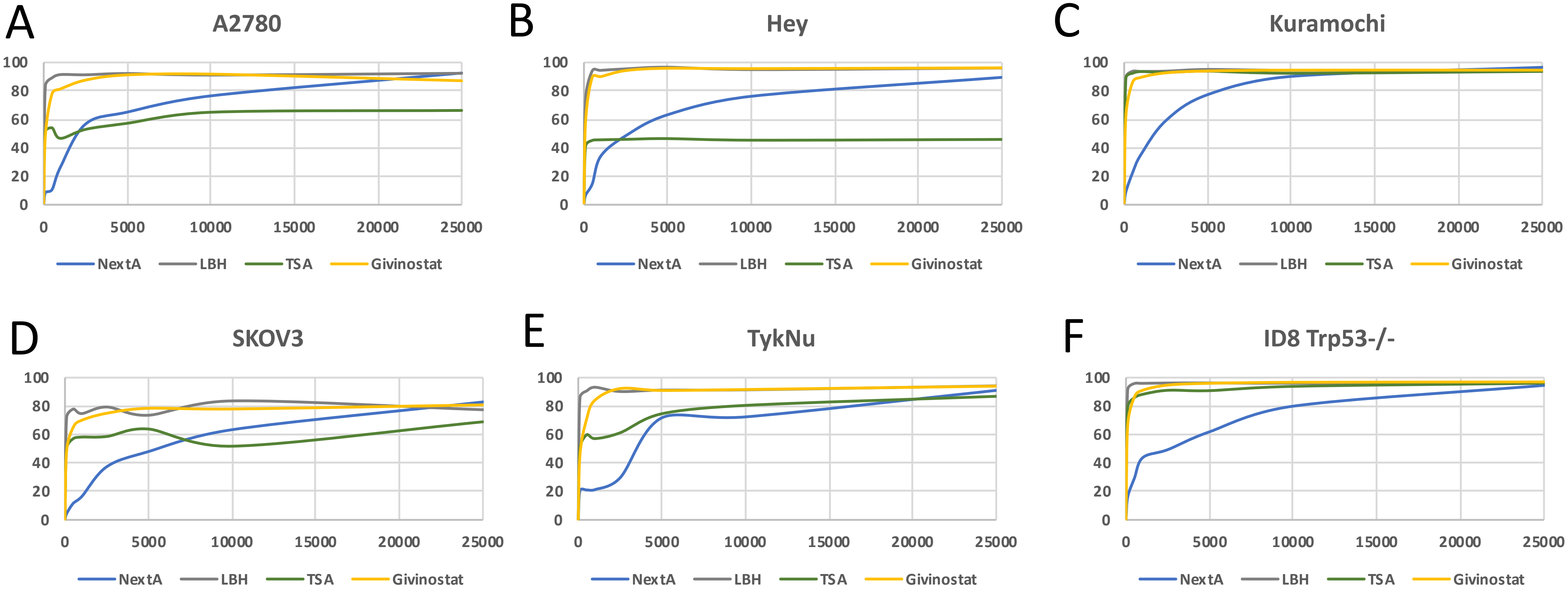
A
A2780
B
Hey
C
Kuramochi
D
SKOV3
E
TykNu
F
ID8 Trp53-/-
0
20
40
60
80
100
0
5000
10000
15000
20000
25000
NextA
LBH
TSA
Givinostat

Figure S2

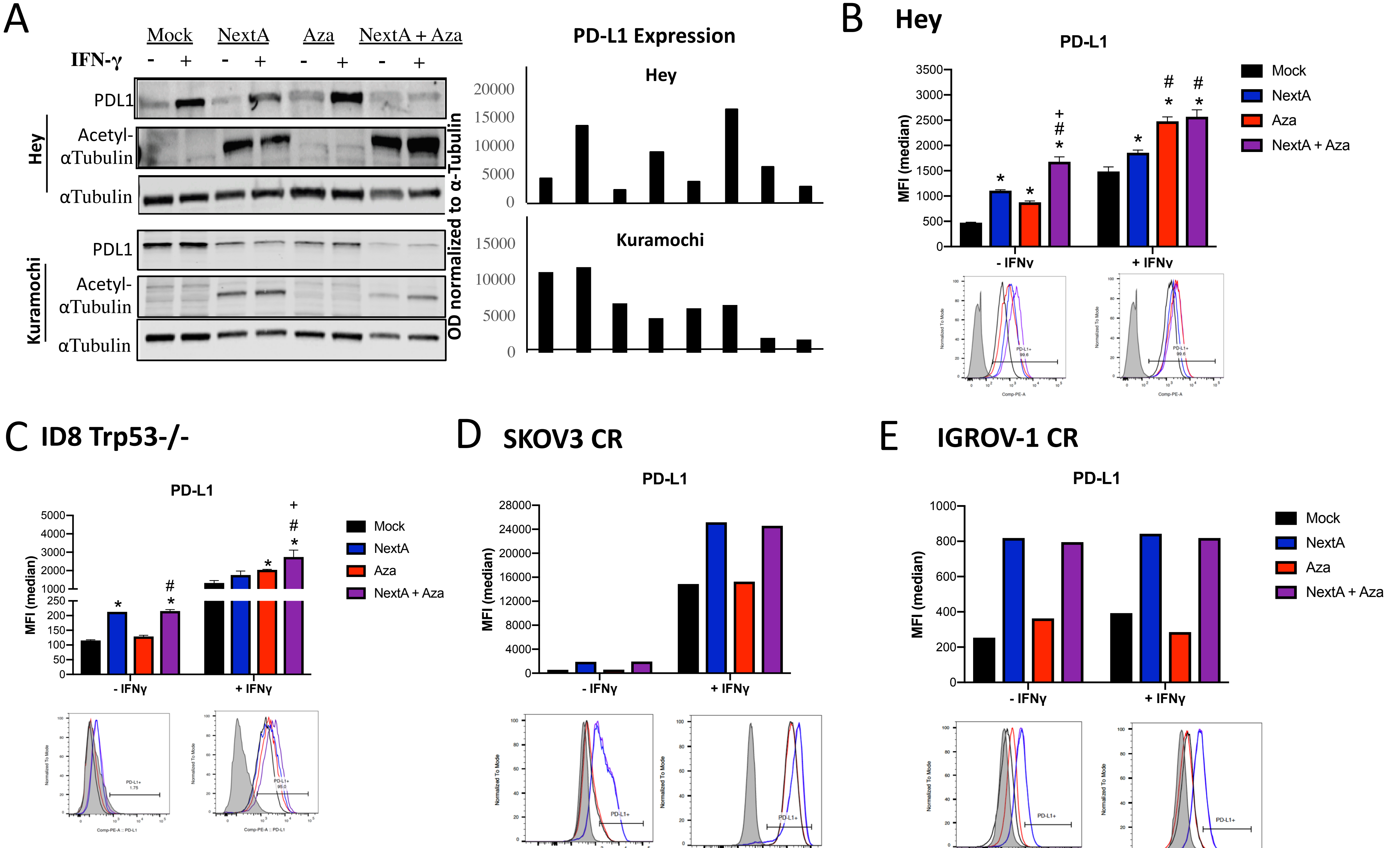
A
Mock
NextA
Aza
NextA + Aza
IFN-γ
PDL1
Acetyl-αTubulin
αTubulin
Hey
Kuramochi
PD-L1 Expression
OD normalized to α-Tubulin
B
Hey
PD-L1
MFI (median)
- IFNγ
+ IFNγ
C
ID8 Trp53-/-
D
SKOV3 CR
E
IGROV-1 CR

Figure S3

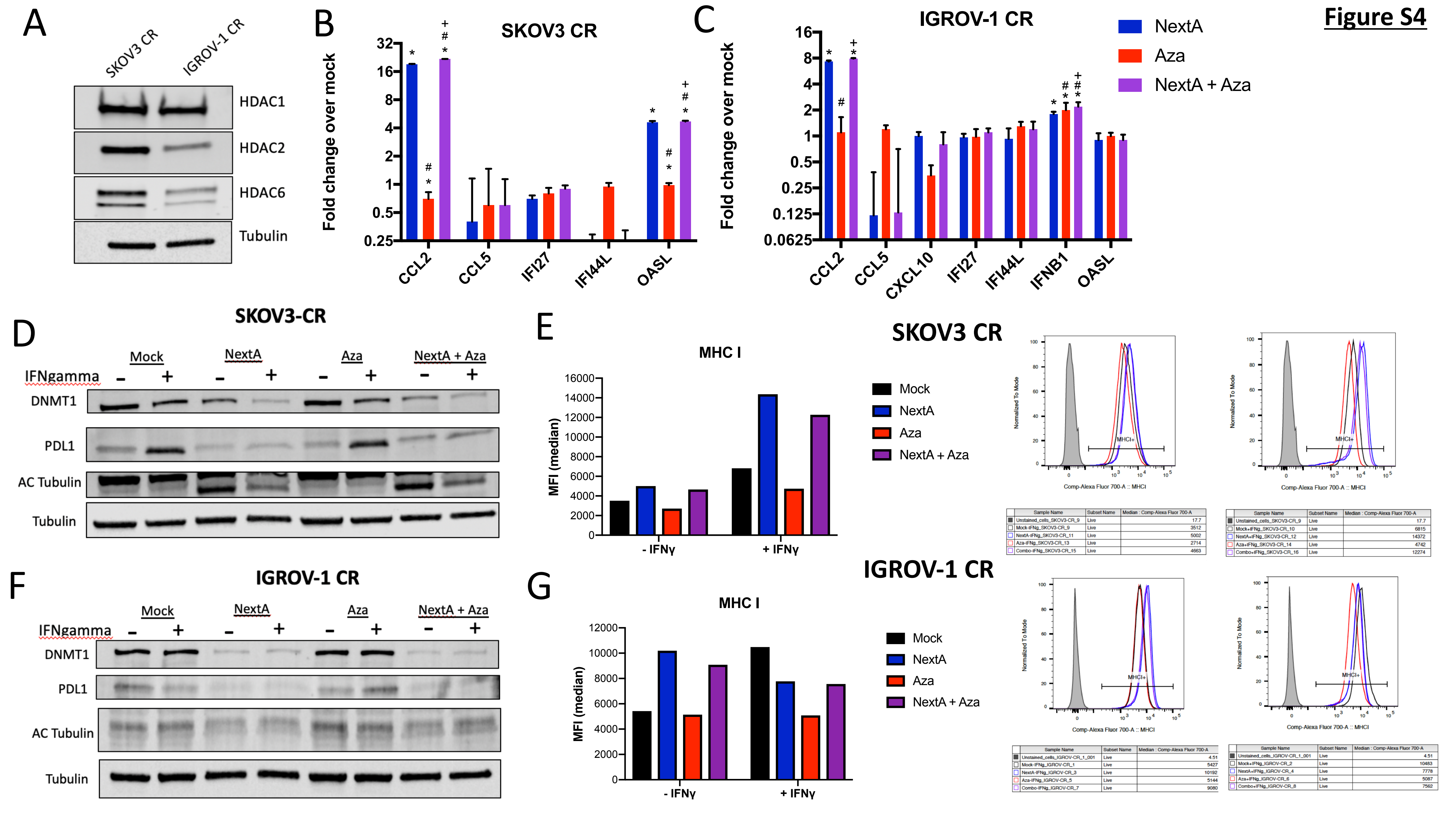
Figure S4
A
SKOV3 CR
IGROV-1 CR
HDAC1
HDAC2
HDAC6
Tubulin
B
SKOV3 CR
Fold change over mock
32
16
8
4
2
1
0.5
0.25
CCL2
CCL5
IFI27
IFI44L
OASL
C
IGROV-1 CR
Fold change over mock
16
8
4
2
1
0.5
0.25
0.125
0.0625
CCL2
CCL5
CXCL10
IFI27
IFI44L
IFNB1
OASL
NextA
Aza
NextA + Aza
D
SKOV3-CR
Mock
NextA
Aza
NextA + Aza
IFNgamma
DNMT1
PDL1
AC Tubulin
Tubulin
E
MHC I
MFI (median)
16000
14000
12000
10000
8000
6000
4000
2000
0
- IFNγ
+ IFNγ
Mock
NextA
Aza
NextA + Aza
SKOV3 CR
Normalized To Mode
MHCI+
Comp-Alexa Fluor 700-A :: MHCI
F
IGROV-1 CR
Mock
NextA
Aza
NextA + Aza
IFNgamma
DNMT1
PDL1
AC Tubulin
Tubulin
G
MHC I
MFI (median)
12000
10000
8000
6000
4000
2000
0
- IFNγ
+ IFNγ
Mock
NextA
Aza
NextA + Aza
IGROV-1 CR
Normalized To Mode
MHCI+
Comp-Alexa Fluor 700-A :: MHCI

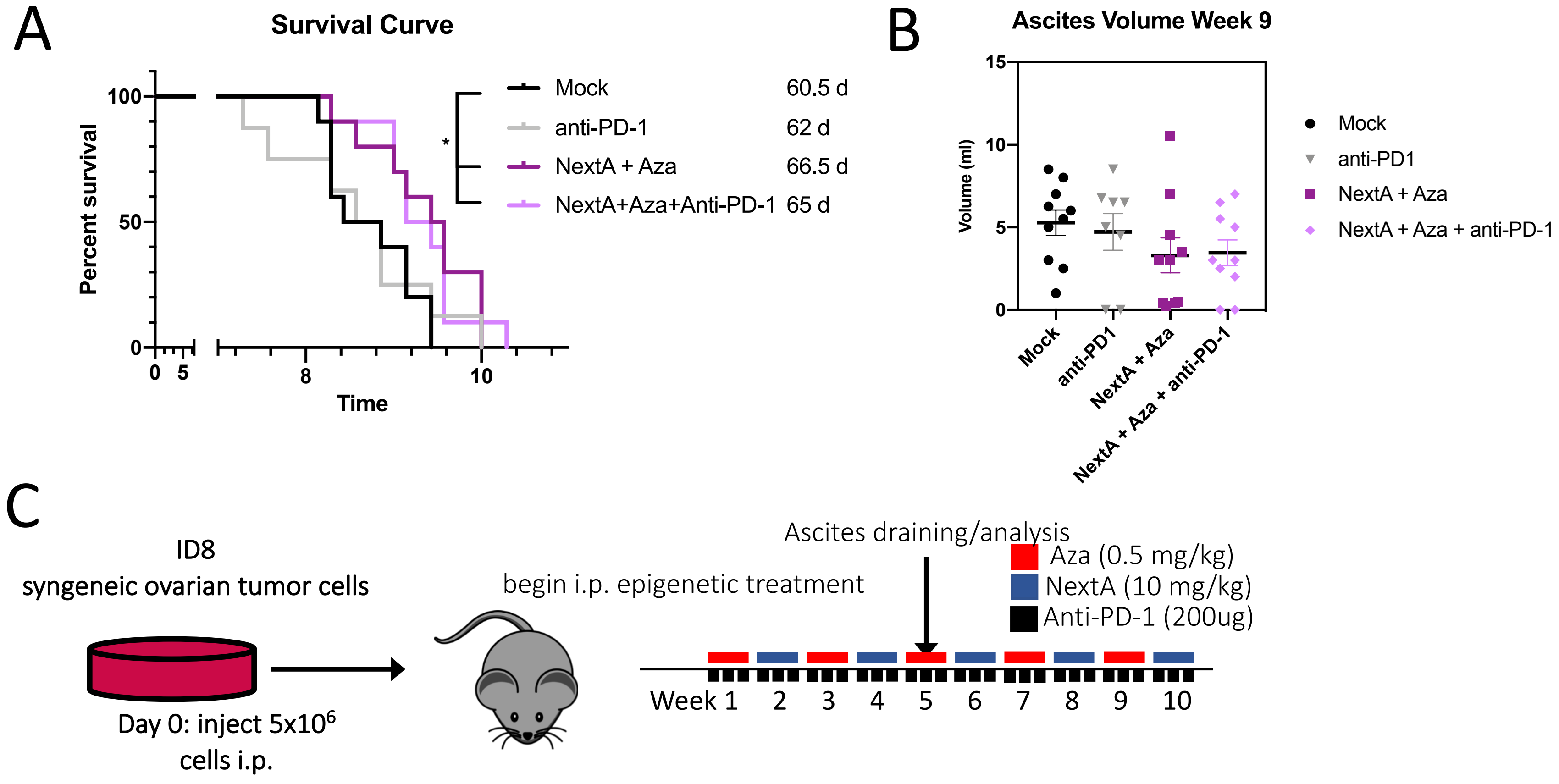
A
Survival Curve
Percent survival
100
50
0
0
5
8
10
Time
*
Mock 60.5 d
anti-PD-1 62 d
NextA + Aza 66.5 d
NextA+Aza+Anti-PD-1 65 d
B
Ascites Volume Week 9
Volume (ml)
15
10
5
0
Mock
anti-PD1
NextA + Aza
NextA + Aza + anti-PD-1
Mock
anti-PD1
NextA + Aza
NextA + Aza + anti-PD-1
C
ID8
syngeneic ovarian tumor cells
Day 0: inject 5x10^6 cells i.p.
begin i.p. epigenetic treatment
Ascites draining/analysis
Aza (0.5 mg/kg)
NextA (10 mg/kg)
Anti-PD-1 (200ug)
Week 1
2
3
4
5
6
7
8
9
10

Figure S5

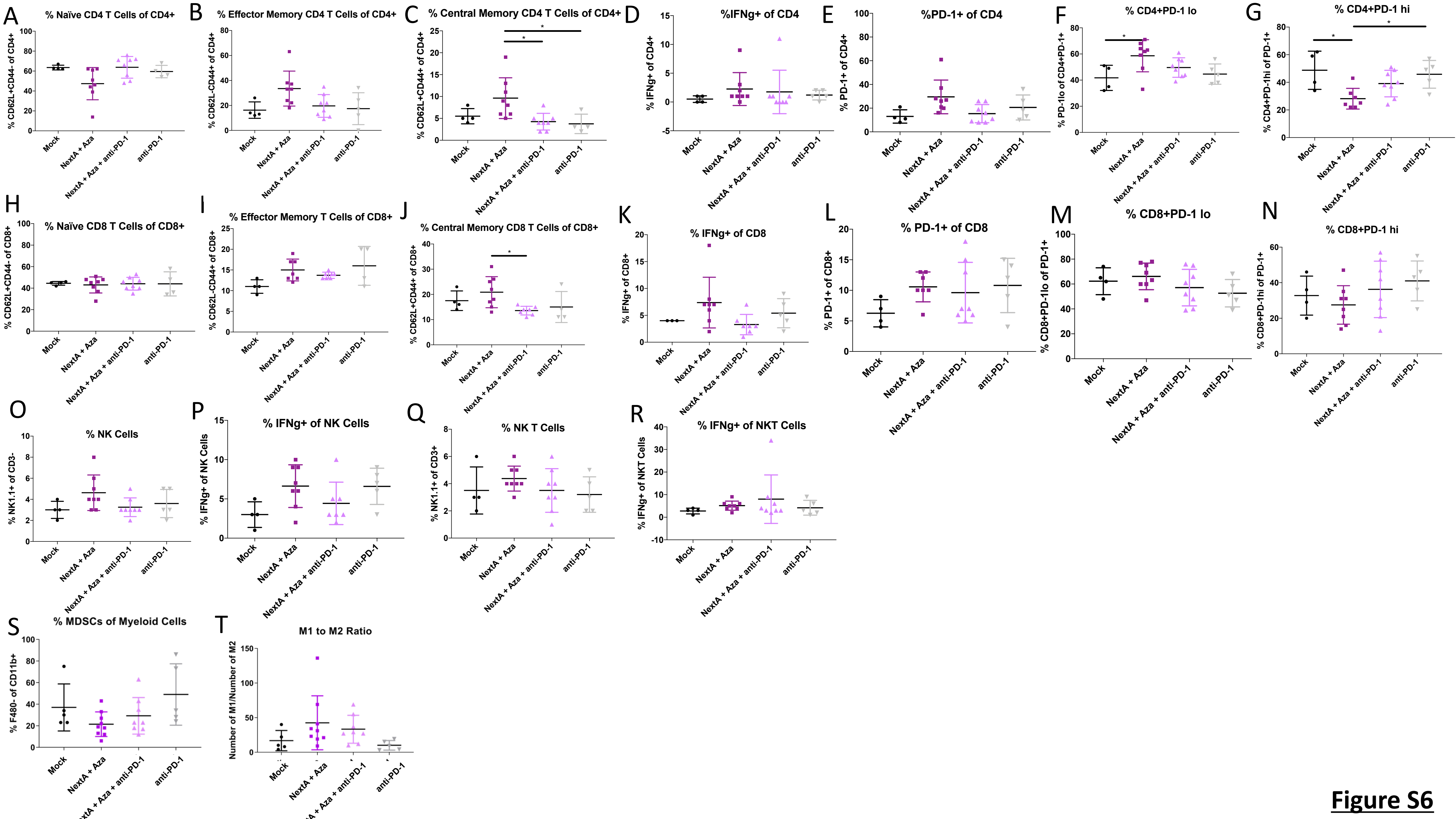
A % Naïve CD4 T Cells of CD4+
B % Effector Memory CD4 T Cells of CD4+
C % Central Memory CD4 T Cells of CD4+
D %IFNg+ of CD4
E %PD-1+ of CD4
F % CD4+PD-1 lo
G % CD4+PD-1 hi
H % Naïve CD8 T Cells of CD8+
I % Effector Memory T Cells of CD8+
J % Central Memory CD8 T Cells of CD8+
K % IFNg+ of CD8
L % PD-1+ of CD8
M % CD8+PD-1 lo
N % CD8+PD-1 hi
O % NK Cells
P % IFNg+ of NK Cells
Q % NK T Cells
R % IFNg+ of NKT Cells
S % MDSCs of Myeloid Cells
T M1 to M2 Ratio
Mock
NextA + Aza
NextA + Aza + anti-PD-1
anti-PD-1

Figure S6

A **Figure S7**

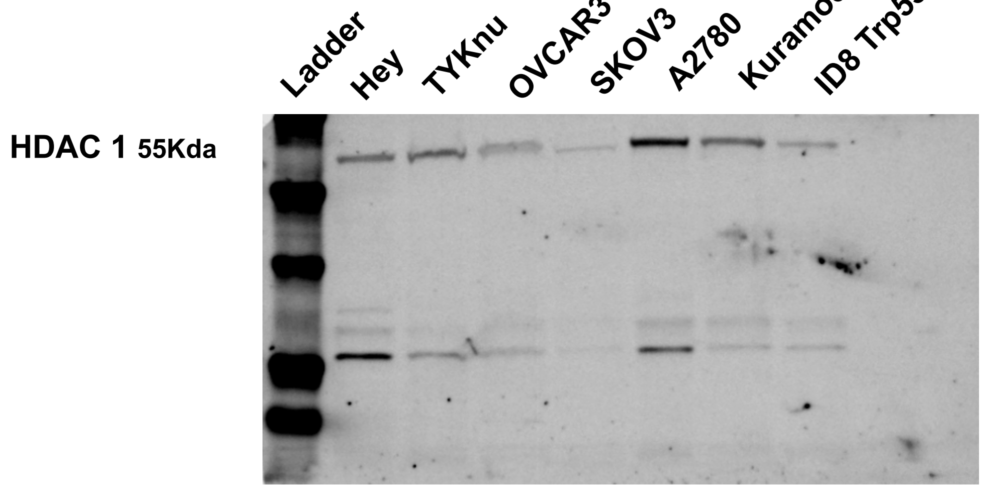

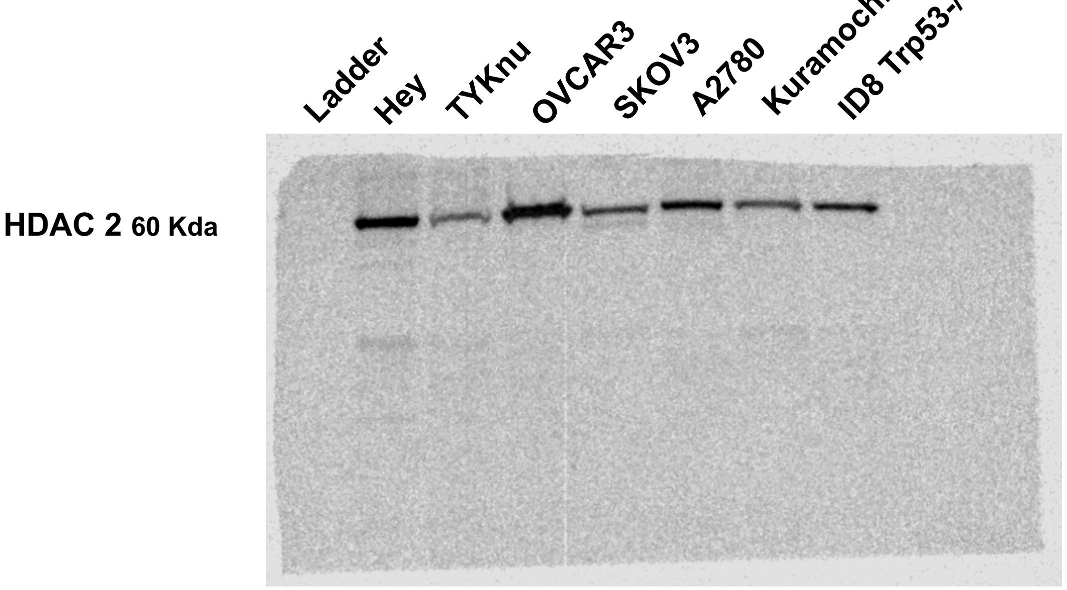

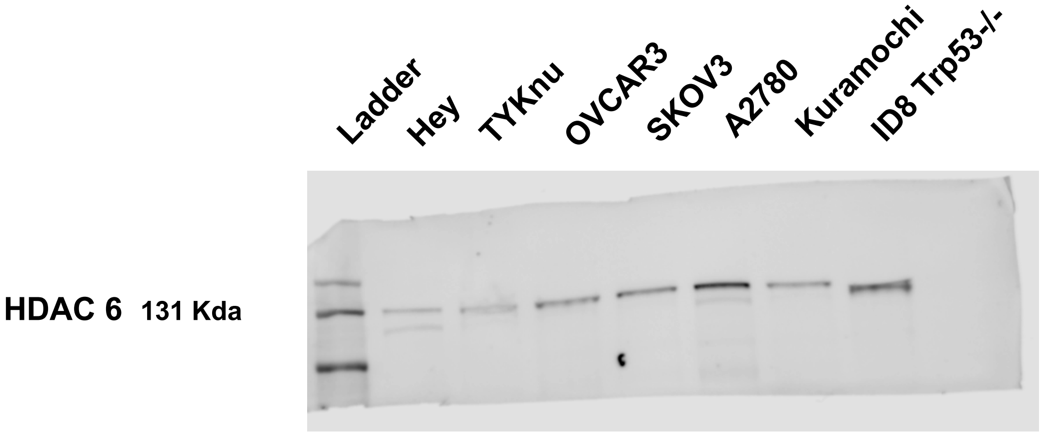

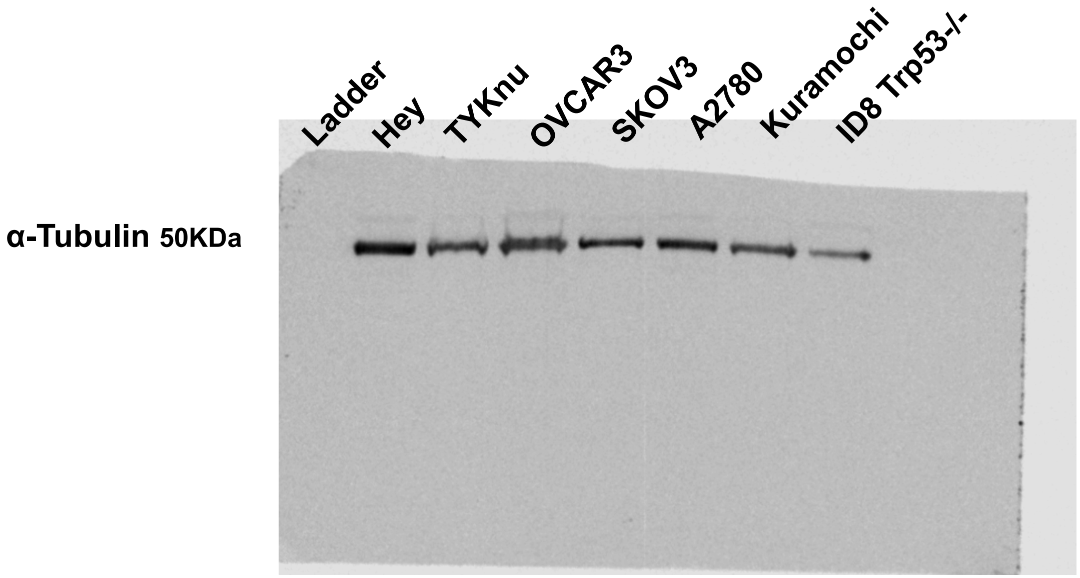

Full blot images from Figure 1A

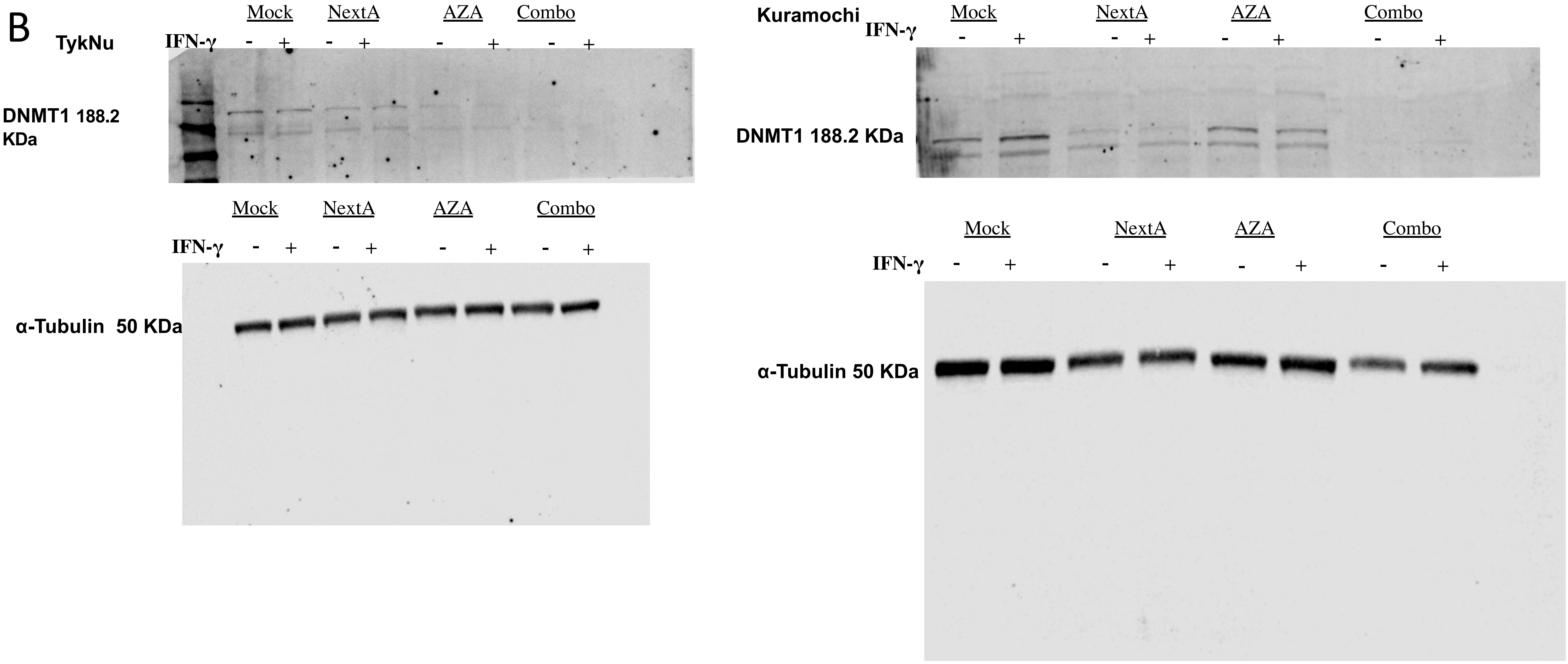

Figure S7

Full blot images from Figure 3A

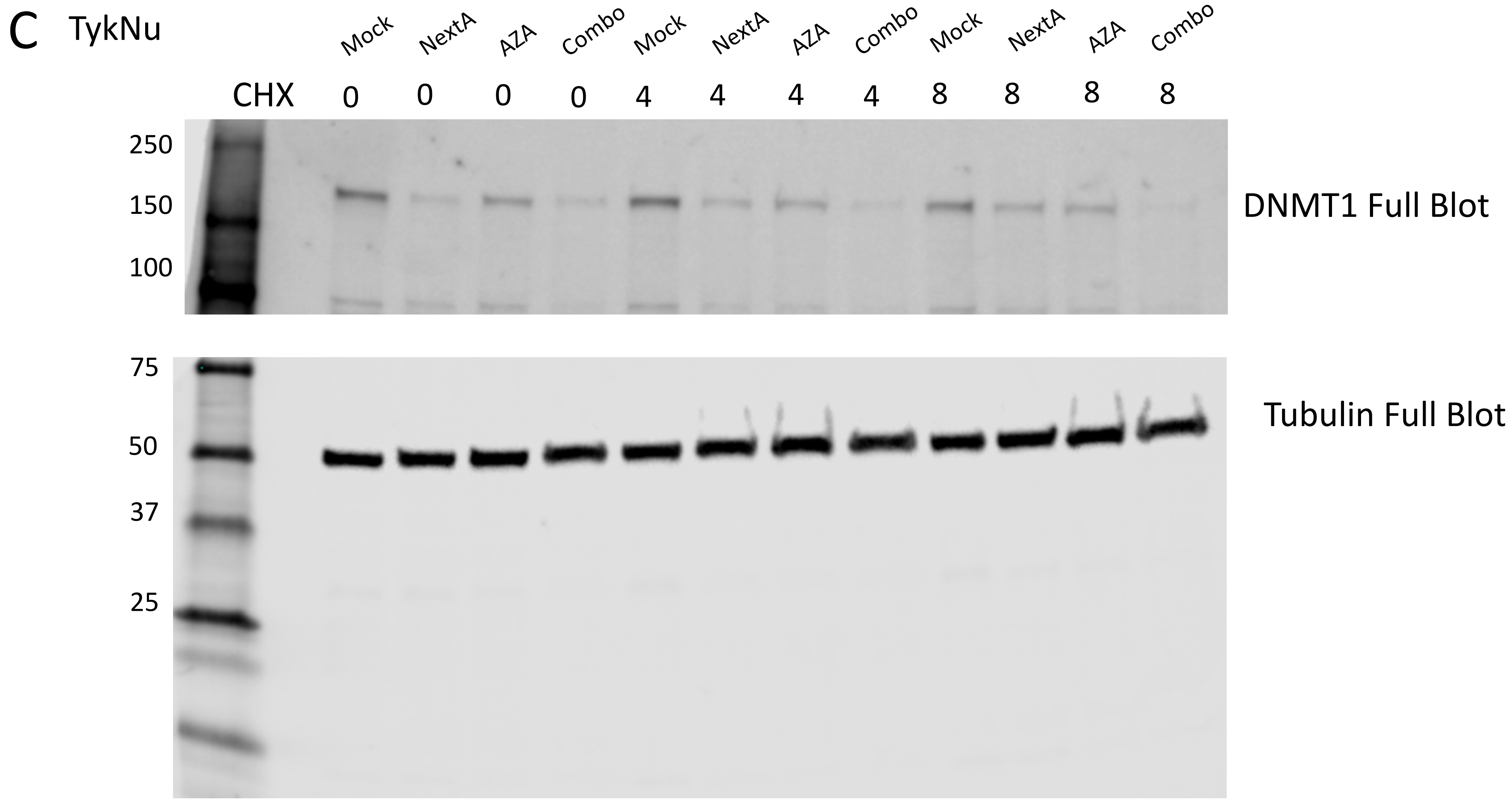

Figure S7

Full blot images from Figure 3B

D

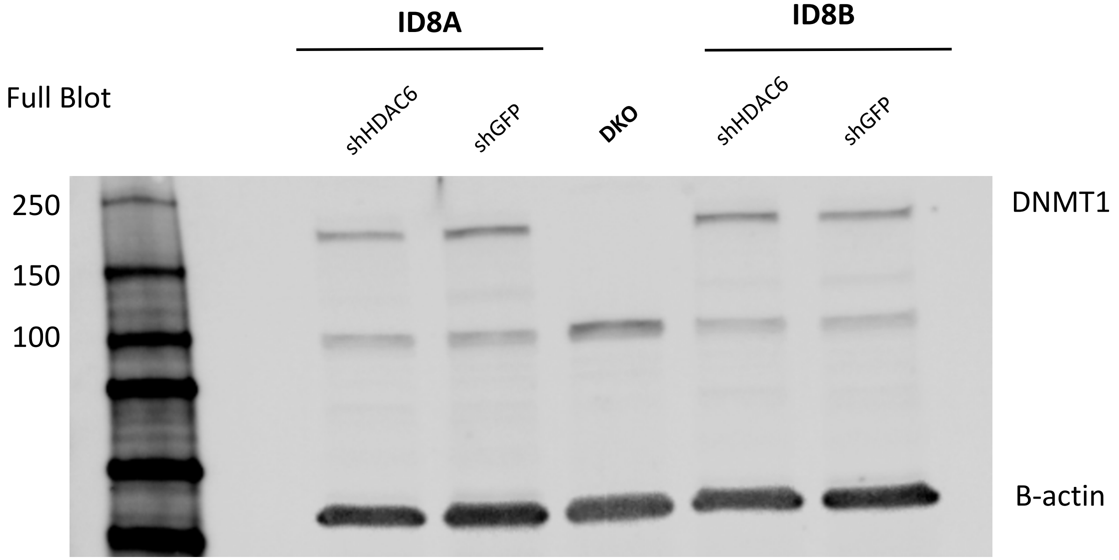

Figure S7

Full blot images from Figure 3C

E

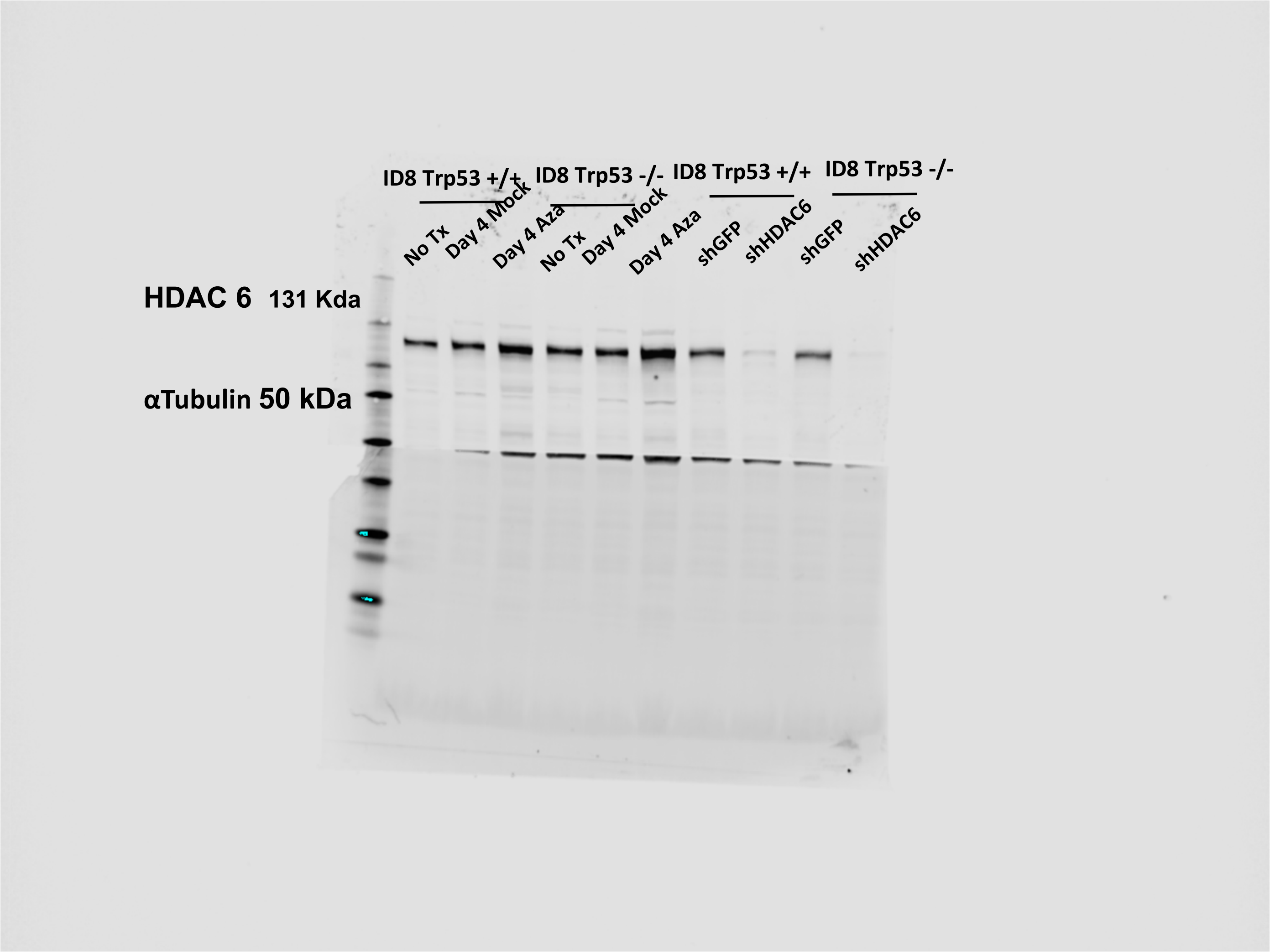

Figure S7

Full blot images from Figure 3D

F **Figure S7**

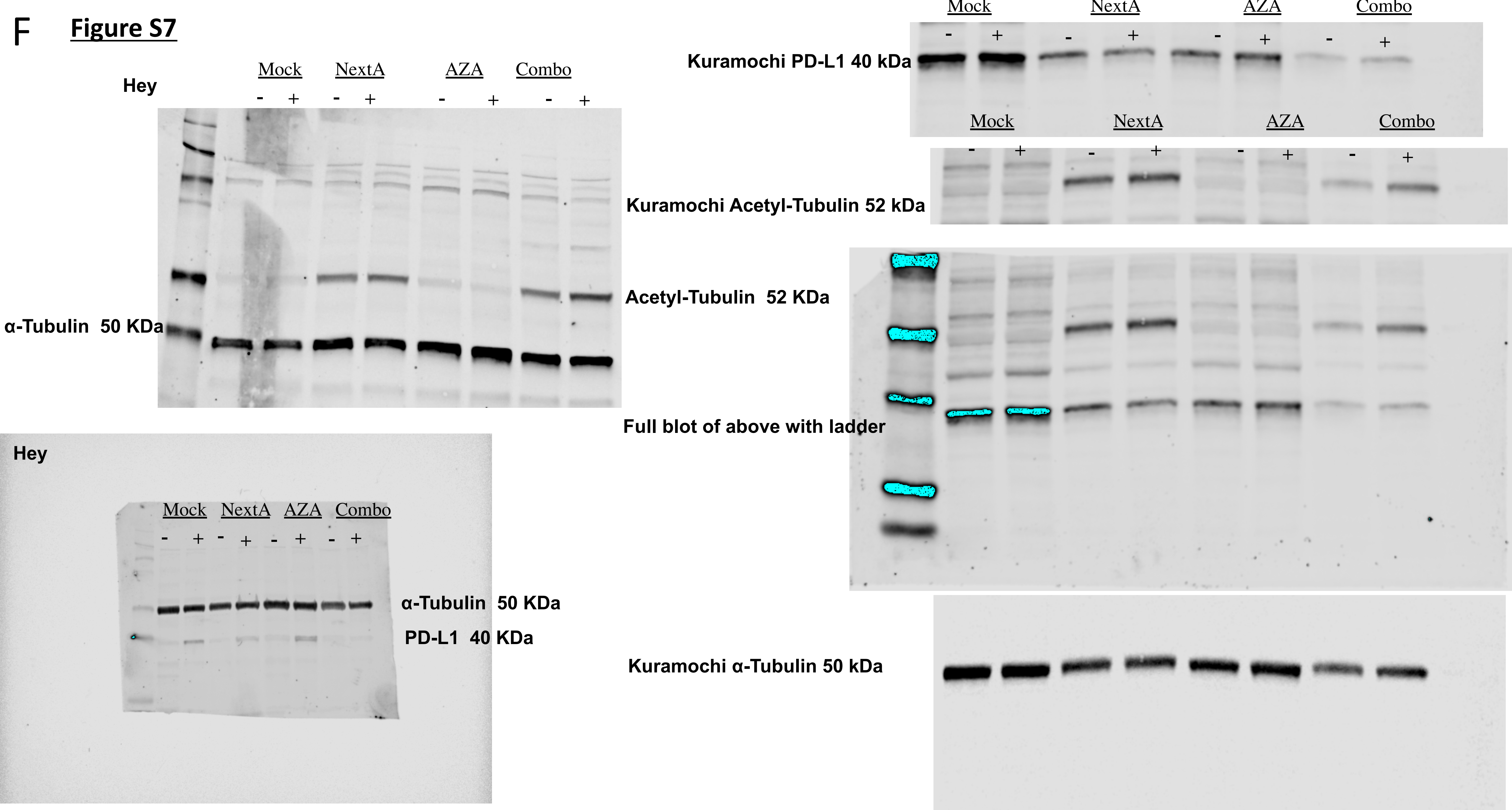

Full blot images from Figure S3A

G **Figure S7**

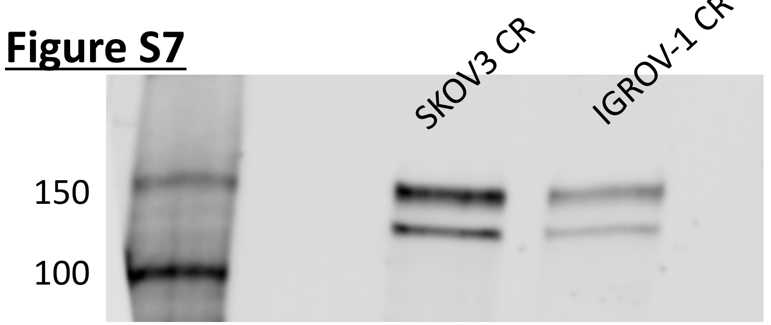

HDAC6 Full Blot

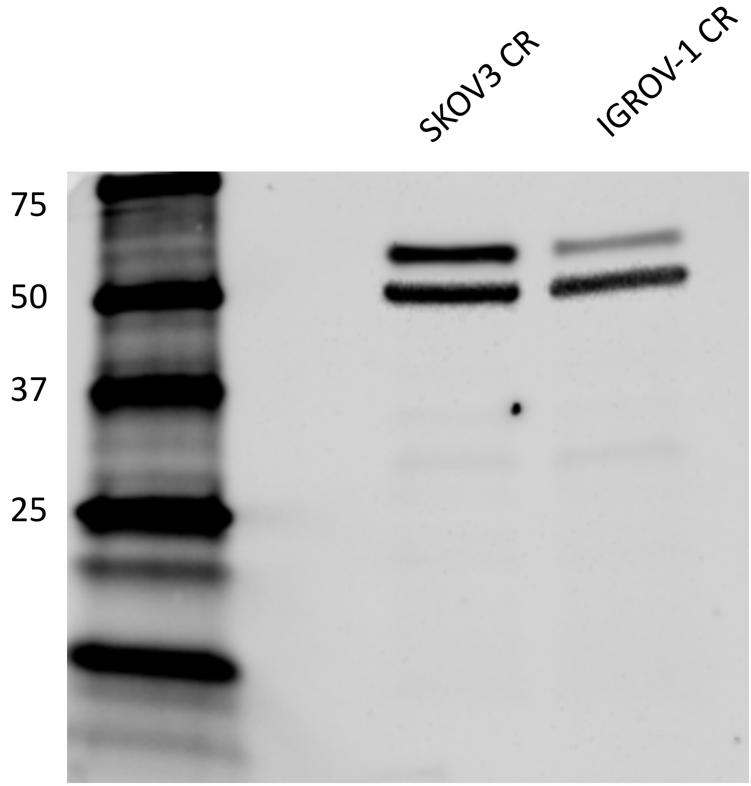

HDAC2 and Tubulin Full Blot

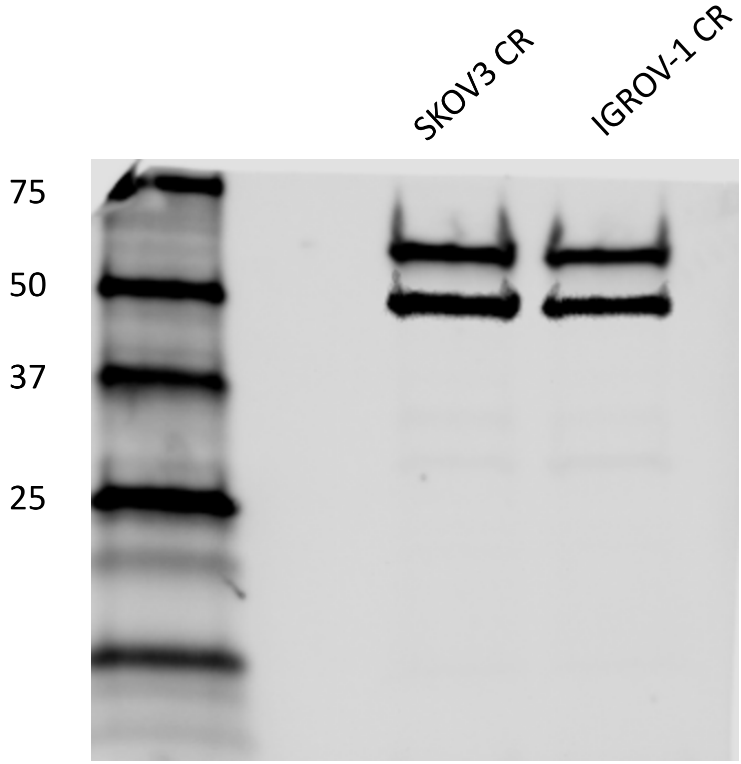

HDAC1 and Tubulin Full Blot

Full blot images from Figure S4A

H **Figure S7**

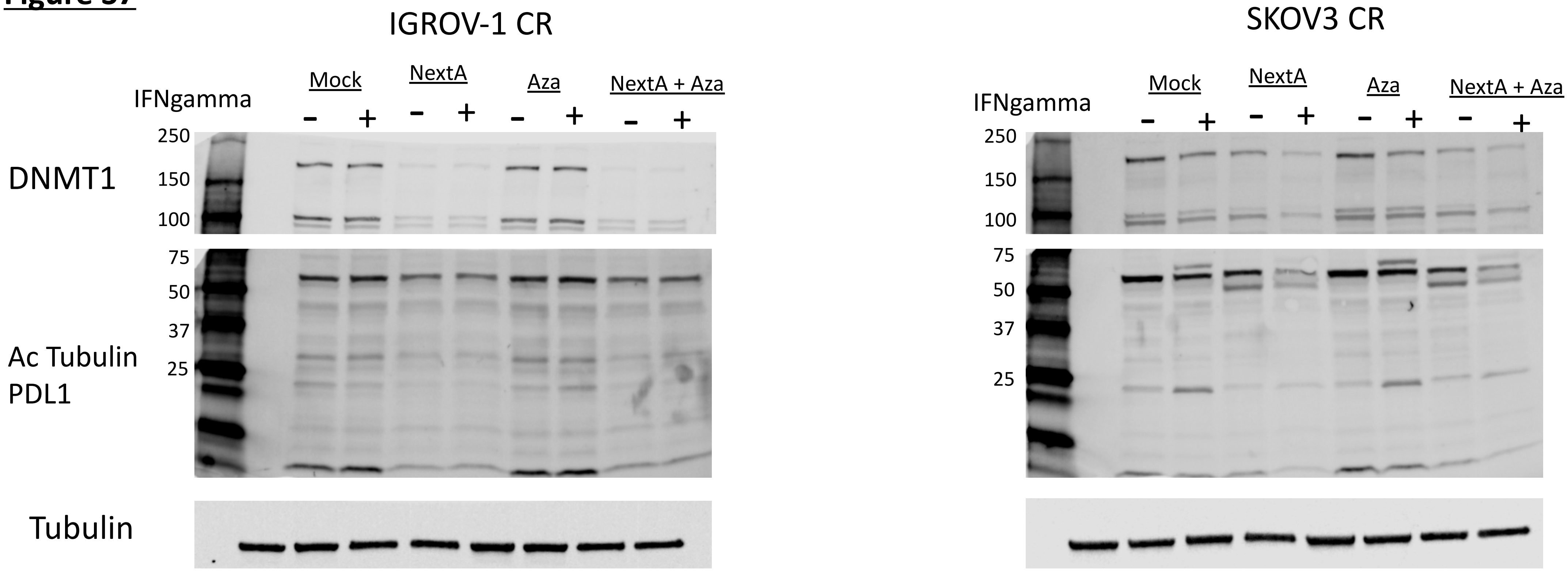

Full blot images from Figure S4D, S4F
